# EPAC1 enhances brown fat growth and beige adipogenesis

**DOI:** 10.1038/s41556-023-01311-9

**Published:** 2024-01-09

**Authors:** Laia Reverte-Salisa, Sana Siddig, Staffan Hildebrand, Xi Yao, Jelena Zurkovic, Michelle Y. Jaeckstein, Joerg Heeren, Frank Lezoualc’h, Natalie Krahmer, Alexander Pfeifer

**Affiliations:** 1 Institute of Pharmacology and Toxicology, University Hospital, University of Bonn, Bonn, Germany; 2 Institute of Biochemistry and Molecular Cell Biology, University Medical Center Hamburg-Eppendorf, Hamburg, Germany; 3 Institute of Cardiovascular and Metabolic Diseases, Inserm UMR-1297, Université Toulouse -Paul Sabatier, Toulouse, France; 4 Institute for Diabetes and Obesity, Helmholtz Center Munich, Neuherberg, Germany; 5 PharmaCenter Bonn, University of Bonn, Bonn, Germany

**Keywords:** Fat metabolism, Mechanisms of disease

## Abstract

Brown adipose tissue (BAT) is a central thermogenic organ that enhances energy expenditure and cardiometabolic health. However, regulators that specifically increase the number of thermogenic adipocytes are still an unmet need. Here, we show that the cAMP-binding protein EPAC1 is a central regulator of adaptive BAT growth. In vivo, selective pharmacological activation of EPAC1 increases BAT mass and browning of white fat, leading to higher energy expenditure and reduced diet-induced obesity. Mechanistically, EPAC1 coordinates a network of regulators for proliferation specifically in thermogenic adipocytes, but not in white adipocytes. We pinpoint the effects of EPAC1 to PDGFRα-positive preadipocytes, and the loss of EPAC1 in these cells impedes BAT growth and worsens diet-induced obesity. Importantly, EPAC1 activation enhances the proliferation and differentiation of human brown adipocytes and human brown fat organoids. Notably, a coding variant of *RAPGEF3* (encoding EPAC1) that is positively correlated with body mass index abolishes noradrenaline-induced proliferation of brown adipocytes. Thus, EPAC1 might be an attractive target to enhance thermogenic adipocyte number and energy expenditure to combat metabolic diseases.

## Abstract (web summary)

Reverte-Salisa et al. show that, in preadipocytes, EPAC1 enhances brown adipose tissue growth and increases the function of thermogenic fat in obesogenic conditions. Activation of EPAC1 induces human brown adipocyte proliferation and differentiation.

## Main

BAT dissipates energy mainly through uncoupling protein 1 (UCP1) (by non-shivering thermogenesis)^1–3^. Increased BAT mass is correlated with leanness and with a decreased risk of cardiovascular disease in human adults^4–6^. In addition to brown adipocytes, another type of thermogenic adipocyte, beige cells, can be found mainly in the subcutaneous white adipose tissue (WAT). These beige adipocytes can be induced by cold exposure or pharmacologically, in a process called browning/beiging^7,8^.

3′,5′-cyclic adenosine monophosphate (cAMP) is the master regulator of brown and beige adipocytes^3,9^. Activation of G_s_-coupled β-adrenergic or purinergic receptors induces cAMP production^3,10^. To date, the vast majority of studies on cAMP signalling focused on protein kinase A (PKA), which mediates cAMP-induced activation of lipolysis and UCP1-dependent energy expenditure^7,9^. Apart from PKA, cAMP can also signal through the exchange proteins directly activated by cAMP (EPAC)^11^. EPAC proteins act as guanine nucleotide exchange factors, and have a similar affinity to cAMP as PKA^12^. EPAC proteins also have a role in the central regulation of leptin^13^, as well as in the secretion of insulin in pancreatic β-cells^14^ and the phosphorylation of AKT in skeletal muscle^15^.

In this Article, we show through genetic and pharmacological manipulation of EPAC1 signalling that EPAC1 regulates thermogenic progenitors and energy balance. Mechanistically, EPAC1 is functionally distinct from the classical PKA signalling pathway and specifically enhances brown fat growth and beige adipogenesis. Finally, we demonstrate that this role of EPAC1 is conserved in human thermogenic adipocytes and that a loss-of-function mutation, which correlates with body mass index (BMI), prevents noradrenaline (NA)-induced brown preadipocyte proliferation.

## Results

### Expression of EPAC1 in BAT and WAT

**Extended Data Fig. 1:**
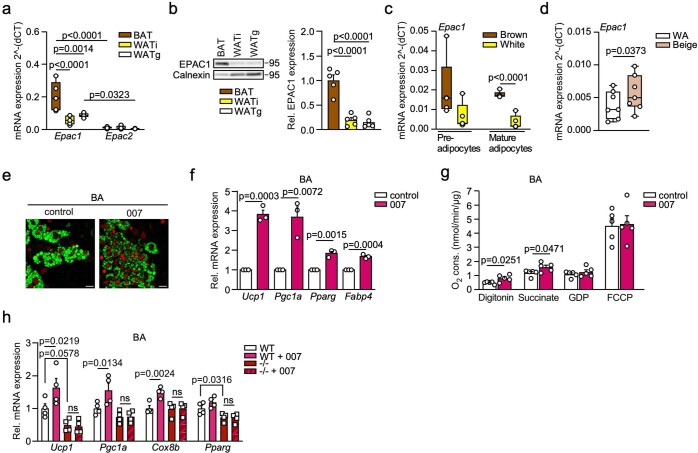
Activation of EPAC1 promotes brown adipogenesis. a, *Epac1* (*Rapgef3*) and *Epac2* (*Rapgef4*) expression in BAT, WATi and WATg of 8 week old mice. n = 5, independent experiments. b, EPAC1 expression in BAT, WATi and WATg. Expression levels normalized to Calnexin, n = 5, biologically independent samples. c, *Epac1 (Rapgef3)* expression in pre- and mature brown versus white adipocytes. n = 5 independent experiments. d, *Epac1 (Rapgef3)* expression of white adipocytes vs white adipocytes beiged after sustained treatment with NE (norepinephrine/noradrenaline), n = 7 biologically independent samples. e, Brown adipocytes (BA) stained with LipidTOX (green) for lipid droplet accumulation after being treated ± 007 throughout differentiation. Scale bar 20 µm. f, relative mRNA expression of BA differentiation markers, n = 3 independent experiments. g, *In vitro* oxygen consumption of mature BA ± 007 throughout differentiation, n = 5 biologically independent samples. h, Relative mRNA expression of BA differentiation markers in primary BA isolated from WT and *Rapgef3*^-/-^ mice and stimulated throughout differentiation ± 007, n = 4 biologically independent samples. P values determined by unpaired two-tailed t-test. In a, two-way ANOVA (Tukey’s multiple comparison test), in d two-tailed paired t-test, in b, h, one-way ANOVA (Tukey’s multiple comparison test). Data are presented as mean values ± SEM. For the box plots in a, c and d the centre line shows the median, the box limits show the first and third quartiles, the upper and lower whiskers extend from the maximum to the minimum value. Source numerical data and unprocessed blots are available in source data. Source data

Analysis of *Rapgef3* and *Rapgef4* (encoding EPAC1 and EPAC2, respectively) expression in different adipose tissue depots revealed that the predominant isoform in adipose tissues was *Rapgef3*, and that BAT expressed the highest levels of EPAC1 (Extended Data Fig. 1a,b). *Rapgef3* was also more highly expressed in brown versus white preadipocytes and mature adipocytes (Extended Data Fig. 1c). Moreover, beige adipocytes expressed significantly more *Rapgef3* compared with white adipocytes (Extended Data Fig. 1d), indicating that EPAC1 might have a major role in brown/beige adipocytes.

### EPAC1 enhances differentiation of brown adipocytes

**Fig. 1:**
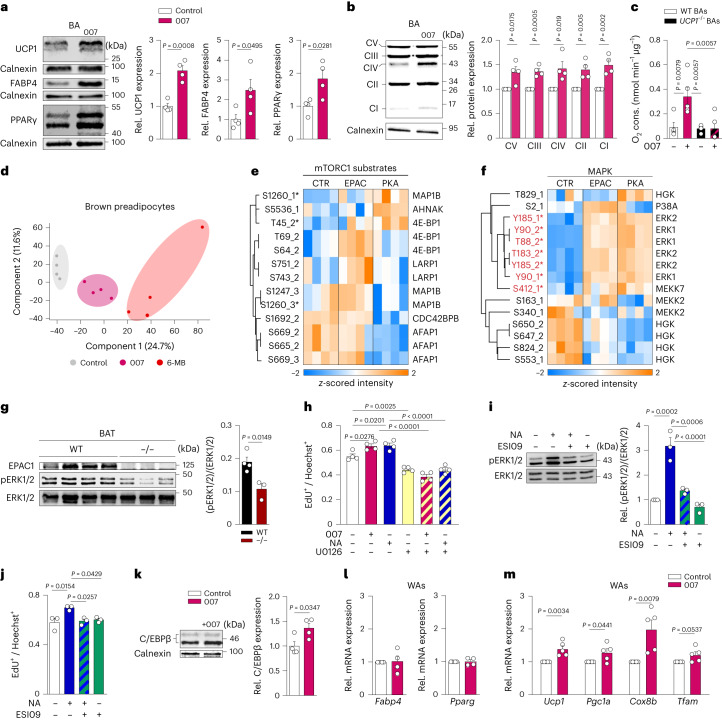
Activation of EPAC1 promotes brown adipogenesis. **a**–**c**, Brown adipocytes (BAs) were differentiated in the presence and absence of 007 during differentiation (from day −2 to day 7). **a**, Representative immunoblot (left) and quantification (right) of UCP1, FABP4 and PPARγ*.* *n* = 4 independent experiments. **b**, Representative immunoblot (right) and quantification (left) of mitochondrial complex components. *n* = 4 independent experiments. **c**, Quantification of UCP1-dependent respiration of WT and *Ucp1*^*−/−*^ brown adipocytes with or without 007. *n* = 5 (WT) and *n* = 3 (*Ucp1*^*−/−*^) independent experiments. Cons., consumption. **d**, Principal component analysis showing different phosphoproteomic signatures in control-, 007- and 6-MB-treated samples. *n* = 4 biologically independent samples. **e**,**f**, Supervised hierarchical clustering of mTORC1 substrates (**e**) and MAPK (**f**). The position of phosphorylation and the multiplicity of phosphopeptide are indicated; the asterisks mark regulatory sites (red, activating). *n* = 4 biologically independent samples. **g**, Representative immunoblot analysis of ERK1/2 phosphorylation (left) and quantification (right) of BAT isolated from *Rapgef3*^*−/*^^*−*^ and WT mice. *n* = 4 (WT) and *n* = 3 (*Rapgef3*^*−/*^^*−*^) biologically independent samples. **h**, Quantification of primary mouse proliferating cells that were pretreated with or without U0126 and stimulated with or without 007, or with or without NA. *n* = 4 biologically independent samples. **i**, Representative immunoblot analysis of ERK1/2 phosphorylation (left) and quantification (right) of brown preadipocytes that were pretreated with or without ESI-09 and stimulated with or without NA. *n* = 3 independent experiments. **j**, Quantification of primary mouse proliferating cells that were pretreated with or without ESI-09 and stimulated with or without NA. *n* = 3 biologically independent samples. **k**, Representative immunoblot (left) and quantification (right) of C/EBPβ in brown adipocytes that were induced with or without 007. *n* = 4 independent experiments. **l**,**m**, Quantitative PCR (qPCR) analysis of the relative (rel.) expression of the adipogenic markers *Fabp4* and *Pparg* (**l**) and the thermogenic markers *Ucp1*, *Pgc1a* and *Cox8b* (**m**) of mature white adipocytes (WAs) differentiated with or without 007. n = 4 (l), *n* = 5 (m) biologically independent samples. *P* values were determined using unpaired two-tailed *t*-tests (**a**, **b**, **g** and **k**–**m**) and one-way analysis of variance (ANOVA) with Tukey’s multiple-comparison test (**c**, **h**, **i** and **j**). Data are mean ± s.e.m. Source numerical data and unprocessed blots are provided as source data. Source data

To investigate the role of EPAC1 in brown adipocyte differentiation, we used the selective and preferential activator of EPAC1 8-pCPT-2′-*O*-Me-cAMP (hereafter, 007)^16^. Treatment of preadipocytes isolated from BAT with 007 resulted in an increase in lipid-droplet accumulation (Extended Data Fig. 1e) and induced a significant increase in the thermogenic marker genes *Ucp1* and *Ppargc1a* (also known as *Pgc1a*; Extended Data Fig. 1f). Moreover, expression of the adipogenic marker genes *Fabp4* and *Pparg* was also significantly increased (Extended Data Fig. 1f). EPAC1 activation induced a significant increase in UCP1, FABP4 and PPARγ protein levels (Fig. 1a), and of the mitochondrial OXPHOS complexes (Fig. 1b). Consistent with these results, 007-treated brown adipocytes displayed significantly increased basal and UCP1-dependent respiration, which was absent in *Ucp1*^*−/−*^ brown adipocytes (Fig. 1c and Extended Data Fig. 1g). Importantly, 007 did not elicit significant changes in *Rapgef3*-knockout (KO; *Rapgef3*^*−/*^^*−*^) primary brown adipocytes (Extended Data Fig. 1h), demonstrating the specificity of 007 for EPAC1. Taken together, these data show that stimulation of EPAC1 enhances brown adipogenesis.

### Identification of EPAC1 signalling pathways in preadipocytes

**Extended Data Fig. 2:**
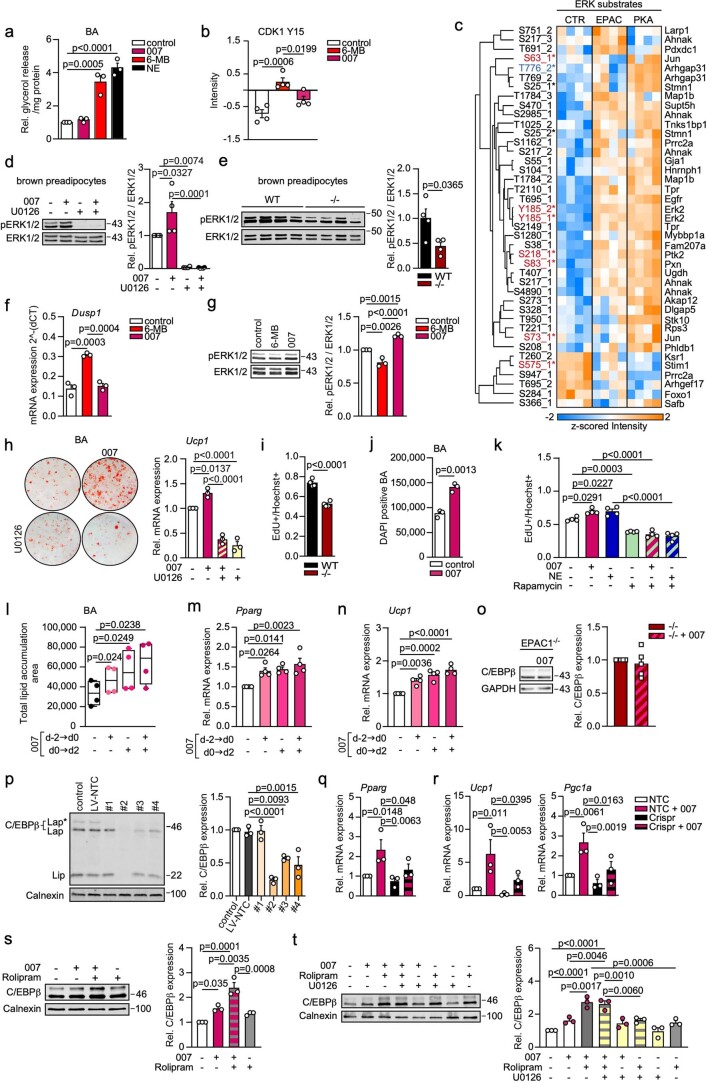
EPAC1 signals through ERK1/2 and upregulates C/EBPβ in brown preadipocytes. **a**, Relative glycerol release of mature BA acutely stimulated with 007, 6-MB or NE (norepinephrine/noradrenaline), n = 3 independent experiments. **b**, Relative CDK1 phosphorylation on Y15 upon 007, 6-MB stimulation, n = 4 biologically independent samples **c**, supervised hierarchical clustering of ERK1/2 substrates. Position of phosphorylation, multiplicity of phosphopeptide indicated, * marks regulatory sites (red =activating, blue=inhibiting). n = 4 biologically independent samples. **d**, Representative immunoblot for ERK1/2 phosphorylation (left panel) and quantification (right panel) of brown preadipocytes stimulated with 007 ± U0126, n = 4 independent experiments. **e**, Representative immunoblot for ERK1/2 phosphorylation (left panel) and quantification (right panel) of brown preadipocytes isolated from *Rapgef3*^-/-^ and WT mice n = 4 biologically independent samples. **f**, *Dusp1* expression in brown preadipocytes upon 007, 6-MB stimulation, n = 3 independent experiments. **g**, Representative immunoblot for ERK1/2 phosphorylation (left panel) and quantification (right panel) of brown preadipocytes stimulated with 007 or 6-MB for 48 h, n = 3 independent experiments. **h**, Oil RedO Staining (left panel) and quantification of *Ucp1* mRNA levels of BA differentiated with 007 ± U0126, n = 3 independent experiments. **i**, Quantification of proliferation of primary murine brown preadipocytes isolated from BAT of *Rapgef3*^-/-^ and WT mice, n = 4 biologically independent samples. **j**, Quantification of DAPI-positive BA after differentiation ± 007 n = 3 independent experiments. **k**, Quantification of primary murine proliferating cells pretreated ± Rapamycin and stimulated ± 007 or ± NE, n = 4 biologically independent samples. **l-n**, BA stimulated during the indicated times with 007. n = 4 independent experiments **l**, Quantification of lipid droplet accumulation of BA. For the box plots, the centre line shows the median, the box limits show the first and third quartiles, the upper and lower whiskers extend from the maximum to the minimum value. **m-n**, relative qPCR expression of the BA differentiation markers *Pparg* and *Ucp1*. **o**, Representative immunoblot (left) and quantification (right) of C/EBPβ in *Rapgef3*^-/-^ BA induced ± 007, n = 5 independent experiments. **p**, Representative immunoblot (left) and quantification (right) of BA transduced with a LV expressing different CRISPR/Cas9 constructs to knock down C/EBPβ, or a non-targeting control construct (LV-NTC), n = 3 biologically independent samples. **q-r**, Relative expression of differentiation markers in BA transduced with LV-NTC or Crispr construct #2 targeting C/EBPβ, and treated with ± 007 throughout differentiation, n = 3 biologically independent samples. **s**, C/EBPβ expression of brown preadipocytes treated with ± 007 ± rolipram, n = 3 independent experiments. **t**, C/EBPβ expression of brown preadipocytes treated with ± 007 ± rolipram ± U0126, n = 3 independent experiments. P values determined by one-way ANOVA (Tukey’s multiple comparison test), unpaired two-tailed t-test in **e, i, j**, and one-way RM ANOVA (Holm-Šídák’s multiple comparison test) in **l-n**. Data are presented as mean values ± SEM. Source numerical data and unprocessed blots are available in source data. Source data

In contrast to stimulation of PKA using either the PKA-specific analogue 6-MB-cAMP (6-MB) or NA, direct activation of EPAC1 did not acutely activate brown adipocytes as measured by lipolysis (Extended Data Fig. 2a). To gain more insights into the differences between EPAC1- and PKA-dependent cAMP signalling in brown preadipocytes, we performed high-sensitivity phosphoproteomics analysis. Principal component analysis demonstrated a clear separation between the two treatments and the control group (Fig. 1d). Specifically, we found that phosphorylation of mTORC1 substrates (4E-BP1 Ser64 and Thr69; LARP1 Ser743/751; and MAP1B Ser1247/1260) was increased after activation of EPAC1, but not PKA (Fig. 1e). mTORC1 is a central controller of anabolic processes in cell growth^17^, suggesting that EPAC1 may have a role in the proliferation of brown preadipocytes. As cell proliferation requires both cellular anabolism and mitosis, we further examined MAPK and CDK1 signalling, which are required for cell cycle entry in response to extracellular cues^18^. High levels of cAMP have previously been shown to block M-phase entry due to inhibitory phosphorylation of CDK1 by PKA at Tyr15 (ref. ^19^). We found that activation of PKA, but not of EPAC1, led to Tyr15 phosphorylation of CDK1 in brown preadipocytes (Extended Data Fig. 2b). Mitogen-activated protein kinase 14 (p38α, Ser2) was phosphorylated only after EPAC1 activation (Fig. 1f). Both EPAC1 and PKA activation induced phosphorylation of ERK1 (Thr88 and Tyr90) and ERK2 (Tyr185 and Thr183) (Fig. 1f), while they had distinct ERK substrate phosphorylation signatures (Extended Data Fig. 2c).

Validation using western blotting showed that 007 significantly increased ERK1/2 phosphorylation, which was completely blunted by the MEK inhibitor U0126 (Extended Data Fig. 2d). Consistent with these findings, preadipocytes and BAT from *Rapgef3*^*−/*^^*−*^ mice showed reduced basal phosphorylation of ERK1/2 compared with the wild type (WT) (Fig. 1g and Extended Data Fig. 2e). To further analyse the differences between EPAC1- and PKA-dependent cAMP signalling, we studied DUSP1, which is known to inhibit ERK signalling by dephosphorylating ERK1/2^20^. *Dusp1* was upregulated after PKA activation (Extended Data Fig. 2f). Consistent with increased *Dusp1* expression, we observed a decrease in ERK1/2 phosphorylation after sustained PKA activation, while the effects of 007 treatment were maintained (Extended Data Fig. 2g).

Physiologically, cold stress stimulates the release of NA and induces BAT growth. As ERK1/2 is activated by NA in several cell types^21^, we first tested whether NA and 007 promote brown preadipocyte proliferation through the MEK/ERK pathway. While 007 and NA increased brown preadipocyte proliferation, this effect was significantly inhibited once the MEK/ERK pathway was blocked (Fig. 1h). Inhibiting ERK1/2 activation with U0126 also blocked the effects of 007 on differentiation (Extended Data Fig. 2h), suggesting that the MEK/ERK pathway has a major role in the EPAC1-dependent effects on brown preadipocyte differentiation. Moreover, NA-induced ERK1/2 phosphorylation was inhibited after blocking EPAC1 using the EPAC inhibitor ESI-09 (Fig. 1i), demonstrating that EPAC1 is required for ERK1/2 activation by NA.

NA-induced proliferation was also completely inhibited by ESI-09 in brown preadipocytes (Fig. 1j). Similarly, *Rapgef3*^*−/*^^*−*^ preadipocytes showed significantly decreased proliferation compared with the WT (Extended Data Fig. 2i). Consistent with this notion, we observed that, at the end of differentiation, the number of lipid-droplet-containing cells was significantly increased after treatment with 007 (Extended Data Fig. 2j).

Notably, inhibition of mTOR by rapamycin also completely blunted the effects of 007 and NA on brown preadipocyte proliferation, suggesting that both ERK1/2 and mTORC1 are downstream mediators of NA-induced proliferation through EPAC1 (Extended Data Fig. 2k).

Taken together, these data strongly indicate that EPAC1 signalling mediates NA-induced proliferation of brown preadipocytes.

To narrow down the timepoint of EPAC1-dependent regulation of differentiation, we stimulated the cAMP/EPAC pathway during different phases of differentiation. Although all treatments significantly increased lipid content and enhanced expression of *Pparg* and *Ucp1*, 007 treatment during both proliferation and the induction phase resulted in the most pronounced effects (Extended Data Fig. 2l-n). To gain further mechanistic insights into additional factors regulating induction, we focused on C/EBPβ, a key transcription factor for brown preadipocyte differentiation that has a crucial role during thermogenic induction^22^ and has been described to be modulated by EPAC1 in tubular epithelial cells^23,24^. Brown preadipocytes treated with 007 during the induction phase exhibited a significant increase in C/EBPβ levels, whereas no effect was observed in *Rapgef3*^*−/*^^*−*^ cells (Fig. 1k and Extended Data Fig. 2o). Knockdown of *Cebpb* (encoding C/EBPβ) using CRISPR–Cas9 (Extended Data Fig. 2p) significantly reduced the effect of 007 on adipogenic and thermogenic markers (Extended Data Fig. 2q,r). To delineate the mechanism of cAMP/EPAC-mediated regulation of C/EBPβ, we focused on phosphodiesterase 4 (PDE4), which is the major PDE regulating cytosolic cAMP in brown preadipocytes^25^, and has also been described to be associated with EPAC1 signalling in other cell types^23,26^. PDE4 inhibition using rolipram enhanced the 007-induced effects on C/EBPβ levels (Extended Data Fig. 2s). These effects were independent of ERK1/2, because blockage of the ERK1/2 signalling pathway showed no effect on the 007-derived upregulation of C/EBPβ expression (Extended Data Fig. 2t).

### EPAC1 enhances browning of white adipocytes

**Extended Data Fig. 3:**
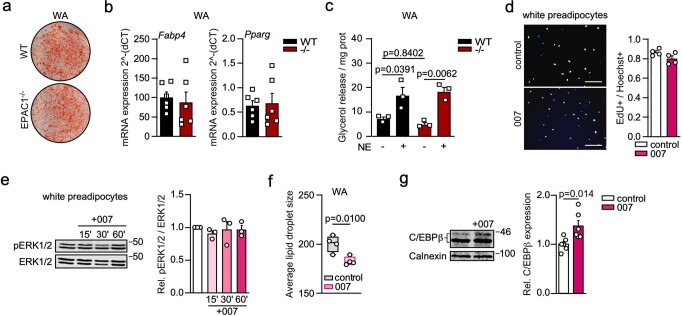
EPAC1 is not required for white preadipocyte proliferation. **a-c**, primary *Rapgef3*^-/-^ and WT white adipocytes (WA). **a**, Oil RedO staining of mature *Rapgef3*^-/-^ and WT WA. **b**, quantification of *Fabp4* and *Pparg* mRNA levels, n = 6 biologically independent samples. **c**, Glycerol release of mature *Rapgef3*^-/-^ and WT WA acutely stimulated with NE (norepinephrine/noradrenaline), n = 3 biologically independent experiments. **d**, Primary murine white preadipocytes stimulated with or without 007 during proliferation. Green: EdU, blue: Hoechst (DNA), n = 4 biologically independent experiments. Scale bar 200 µm. **e**, Representative immunoblot (left) and quantification (right) of rel. ERK1/2 phosphorylation of white preadipocytes stimulated with 007 at the indicated times. Phosphorylation normalized to total ERK1/2 expression, n = 3 biologically independent experiments. **f**, Quantification of average lipid droplet size, n = 4 biologically independent samples. For the box plots, the centre line shows the median, the box limits show the first and third quartiles, the upper and lower whiskers extend from the maximum to the minimum value. **g**, Representative immunoblot (left) and quantification (right) of C/EBPβ in WA induced ± 007, n = 6 biologically independent experiments. P values determined by unpaired two-tailed t-test. In **c** and **e**, one-way ANOVA (Tukey’s multiple comparison test). Data are presented as mean values ± SEM. Source numerical data and unprocessed blots are available in source data. Source data

Stimulation of the cAMP–EPAC1 axis in white preadipocytes did not result in increased *Fabp4* and *Pparg* expression (Fig. 1l). Differentiated *Rapgef3*^*−*^^*/*^^*−*^ white adipocytes showed no difference in adipogenesis or in lipolysis compared with WT cells (Extended Data Fig. 3a–c). Moreover, 007 did not induce any increase in proliferation or ERK1/2 phosphorylation (Extended Data Fig. 3d,e). Thus revealing major differences in the role of EPAC1-dependent regulation between brown and white adipocytes.

By contrast, treatment of white preadipocytes with 007 throughout differentiation resulted in a significant increase in *Ucp1*, *Pgc1a*, *Cox8b* and *Tfam*, alongside a significant decrease in the average lipid-droplet size (Fig. 1m and Extended Data Fig. 3f) and increased C/EBPβ levels (Extended Data Fig. 3g). Taken together, these data show that cAMP enhances the thermogenic program and browning of white adipocytes through EPAC1.

### EPAC1 enhances BAT growth in vivo

**Fig. 2:**
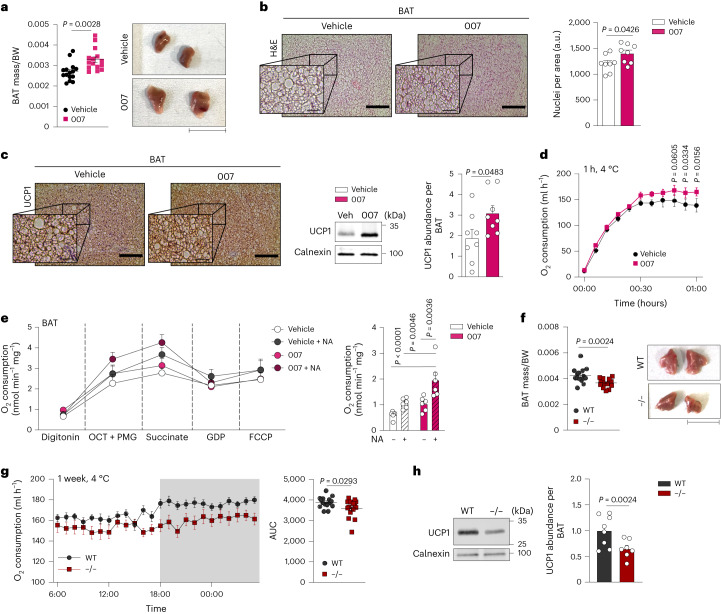
EPAC1 signalling promotes BAT growth and function in vivo. **a**–**e**, Mice were injected daily for 1 week with vehicle or 007 and housed at 23 °C. **a**, BAT mass per body weight (BW; *n* = 12 biologically independent samples; left) and representative macroscopy image (right). Scale bar, 1 cm. **b**, Representative haematoxylin and eosin (H&E) staining of BAT (left). Scale bars, 200 µm (overview) and 25 µm (magnifications). Right, quantification of haematoxylin-stained nuclei per area. *n* = 8 biologically independent samples. a.u., arbitrary units. **c**, Representative immunostaining of UCP1 (left). Scale bars, 200 µm (overview) and 25 µm (magnifications). *n* = 3 biologically independent samples. Right, representative immunoblot and quantification of UCP1 expression in BAT; expression data were normalized to total BAT weight. *n* = 8 independent animals. **d**, The oxygen consumption of mice that were exposed to 4 °C for 1 h. *n* = 4 independent animals. **e**, The oxygen consumption of BAT from mice that were injected with 007 or vehicle and incubated with or without NA (left) and quantification of UCP-1-dependent respiration (right). *n* = 6 biologically independent samples. **f**–**h**, *Rapgef3*^*−/*^^*−*^ and WT mice were housed at 4 °C for 1 week. **f**, BAT mass per body weight (left) and representative macroscopy image. Scale bar, 1 cm (right). *n* = 15 biologically independent samples. **g**, The oxygen consumption of *Rapgef3*^*−/*^^*−*^ and WT mice that were housed for 1 week at 4 °C (left) and quantification of the area under the curve (AUC) (right). *n* = 15 independent animals. **h**, Representative immunoblot and quantification of UCP1 expression in BAT; expression data were normalized to total BAT weight. *n* = 8 biologically independent samples. *P* values were determined using unpaired two-tailed *t*-tests (**a**–**d** and **f**–**h**) and one-way ANOVA with Tukey’s multiple-comparison test (**e**). Data are mean ± s.e.m. Source numerical data and unprocessed blots are provided as source data. Source data

**Extended Data Fig. 4:**
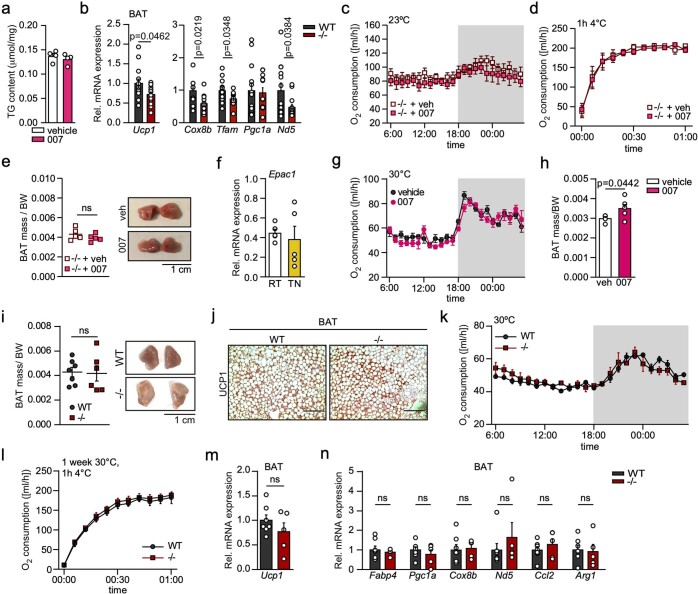
EPAC1 signalling at thermoneutrality. **a**, Triglyceride (TG) content in the BAT of 007 and vehicle treated mice at 23 °C, n = 3 biologically independent samples. **b**, mRNA expression of thermogenic and mitochondrial markers of BAT from EPAC1^-/-^ and WT mice after 1 week at 4 °C, n = 13 biologically independent samples. **c-e**, EPAC1^-/-^ mice injected with NaCl (veh) or 007 for one week. **c**, Oxygen consumption over the course of 24 hours at 23 °C, n = 5 independent animals. **d**, Oxygen consumption of mice exposed for 1 h at 4 °C after 1 week of treatment, n = 5 independent animals. **e**, BAT mass per BW, n = 5 biologically independent samples. **f**, *Epac1 (Rapgef3)* expression in the BAT of mice housed at room temperature (RT, 23 °C) or at thermoneutrality (TN, 30 °C), n = 5 biologically independent samples. **g-h**, Mice housed at 30 °C and injected with 007 or vehicle for a week. **g**, Oxygen consumption over the course of 24 hours at 30 °C, n = 5 (vehicle), n = 6 (007) independent animals. **h**, BAT mass per BW, n = 5 (vehicle), n = 6 (007) biologically independent samples. **i-n**, *Rapgef3*^-/-^ and WT mice housed at 30 °C for 1 week. **i**, BAT mass per BW, n = 7 (WT), n = 6 (*Rapgef3*^-/-^) biologically independent samples. **j**, Representative UCP1 immunostaining of BAT, n = 3 biologically independent samples. Scale bar 100 µm. **k**, Oxygen consumption over the course of 24 hours at 30 °C, n = 4 independent animals. **l**, Oxygen consumption of mice exposed for 1 h at 4 °C after 1 week at 30 °C, n = 4 independent animals. **m-n**, mRNA expression of BAT isolated from *Rapgef3*^-/-^ and WT mice after 1 week at 30 °C, n = 7 (WT), n = 6 (*Rapgef3*^-/-^) biologically independent samples. P values determined by unpaired two-tailed t-test. Data are presented as mean values ± SEM. Source numerical data are available in source data. Source data

To investigate the role of EPAC1 signalling in vivo, we administered 007 daily to adult mice for 1 week, which resulted in a significant increase in BAT mass and cell density, with no significant difference in triglyceride content (Fig. 2a,b and Extended Data Fig. 4a). Concomitantly, expression of UCP1 protein and BAT-dependent energy expenditure were significantly increased (Fig. 2c,d). Furthermore, BAT isolated from 007-injected mice exhibited a significantly enhanced NA-induced UCP1-dependent respiration ex vivo (Fig. 2e).

To further study the physiological relevance of EPAC1, we used whole-body-KO *Rapgef3*^*−/*^^*−*^ mice and exposed them to cold conditions for 1 week. BAT mass was significantly lower in *Rapgef3*^*−/*^^*−*^ mice compared with in WT mice (Fig. 2f). BAT function was significantly reduced in *Rapgef3*^*−/*^^*−*^ mice, which exhibited significantly reduced O_2_ consumption (Fig. 2g) and significantly decreased UCP1 levels and thermogenic markers (*Cox8b*, *Tfam* and *Nd5*) (Fig. 2h and Extended Data Fig. 4b). Notably, *Rapgef3*^*−/*^^*−*^ mice treated with 007 did not exhibit any changes in energy expenditure after short-term cold exposure, and showed no differences in BAT growth (Extended Data Fig. 4c–e), ruling out off-target effects of 007.

Under thermoneutral conditions, *Rapgef3* expression was not changed (Extended Data Fig. 4f). Daily 007 administration at 30 °C induced an increase in BAT mass; however, in the absence of adrenergic stimulus, no significant differences were observed in the oxygen consumption of vehicle- versus 007-treated mice (Extended Data Fig. 4g,h). Similarly, the BAT mass as well as BAT lipid-droplet size of *Rapgef3*^*−/*^^*−*^ mice was comparable to that of the WT mice (Extended Data Fig. 4i,j). Accordingly, we did not observe any differences in O_2_ consumption or in mRNA expression of thermogenic and inflammation markers between *Rapgef3*^*−/*^^*−*^ and WT mice that were housed at thermoneutrality (Extended Data Fig. 4k–n).

These findings suggest that the EPAC1 pathway is crucial for the induction of BAT growth and increased thermogenic capacity after physiological activation/cold exposure.

### Loss of EPAC1 in PDGFRα^+^ cells results in reduced BAT mass

**Fig. 3:**
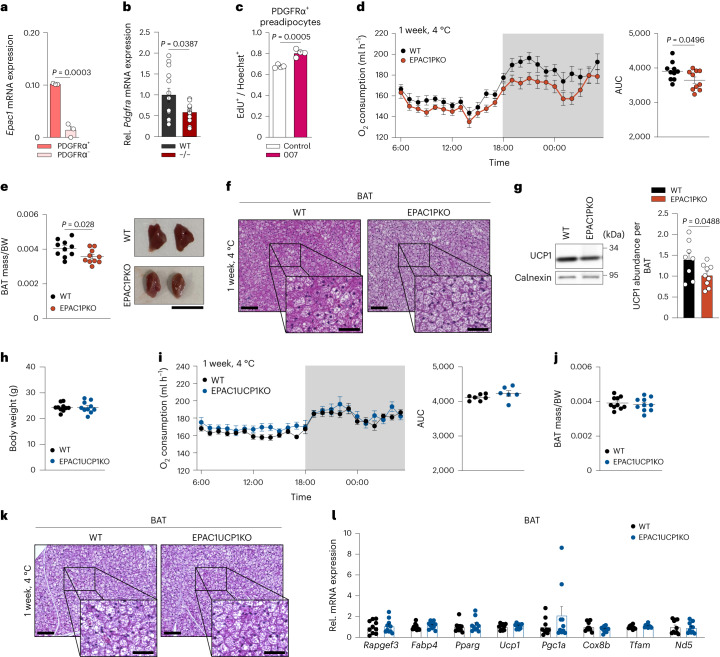
Mice lacking EPAC1 in PDGFRα^+^ cells have decreased cold-induced oxygen consumption and BAT mass. **a**, mRNA expression ($2{^{-\Delta C{_{\mathrm{t}}}}}$) of *Rapgef3* in PDGFRα^+^ versus PDGFRα^−^ cells isolated from BAT. *n* = 3, where each *n* is a pool of 5–6 mice. **b**, *Pdgfra* mRNA expression in BAT isolated from *Rapgef3*^*−/*^^*−*^ and WT mice after 1 week exposure to 4 °C. *n* = 13 biologically independent samples. **c**, Proliferation of primary PDGFRα^+^ cells sorted from BAT and stimulated with or without 007. *n* = 4 biologically independent samples. **d**–**g**, EPAC1PKO and WT (*Rapgef3*^*fl/fl*^*Pdgfra*^*+/+*^) mice were exposed to 4 °C for 1 week. **d**, The oxygen consumption of mice that were exposed for 1 week to 4 °C (left) and quantification of the AUC (right). *n* = 10 independent animals. **e**, The BAT mass per body weight of mice that were exposed for 1 week to 4 °C. *n* = 10 biologically independent samples (left) and representative macroscopy image (right). Scale bar, 1 cm. *n* = 3 biologically independent samples. **f**, Representative H&E immunostaining of BAT after 1 week exposure to 4 °C. Scale bars, 100 µm (overview) and 50 µm (magnifications). *n* = 3 biologically independent samples. **g**, Representative immunoblot (left) and quantification (right) of UCP1 from BAT after 1 week exposure to 4 °C; expression data were normalized to the total BAT weight. *n* = 8 (WT) and *n* = 9 (EPAC1PKO) biologically independent samples. **h**–**l**, EPAC1UCP1KO and WT (*Rapgef3*^*fl/fl*^*Ucp1*^*+/+*^) mice were exposed to 4 °C for 1 week. **h**, The body weight of mice that were exposed for 1 week to 4 °C. *n* = 10 biologically independent samples. **i**, Oxygen consumption of mice that were exposed for 1 week to 4 °C (left) and quantification of the AUC (right). *n* = 7 (WT) and *n* = 6 (EPAC1UCP1KO) independent animals. **j**, The BAT mass per body weight of mice that were exposed for 1 week to 4 °C. *n* = 10 biologically independent samples. **k**, Representative H&E immunostaining of BAT after 1 week exposure to 4 °C. Scale bars, 100 µm (overview) and 50 µm (magnifications). *n* = 3 biologically independent samples. **l**, The mRNA expression profile of BAT from cold-exposed mice. *n* = 10 biologically independent samples. *P* values were determined using unpaired two-tailed *t*-tests (**a**–**l**). Data are mean ± s.e.m. Source numerical data and unprocessed blots are provided as source data. Source data

**Extended Data Fig. 5:**
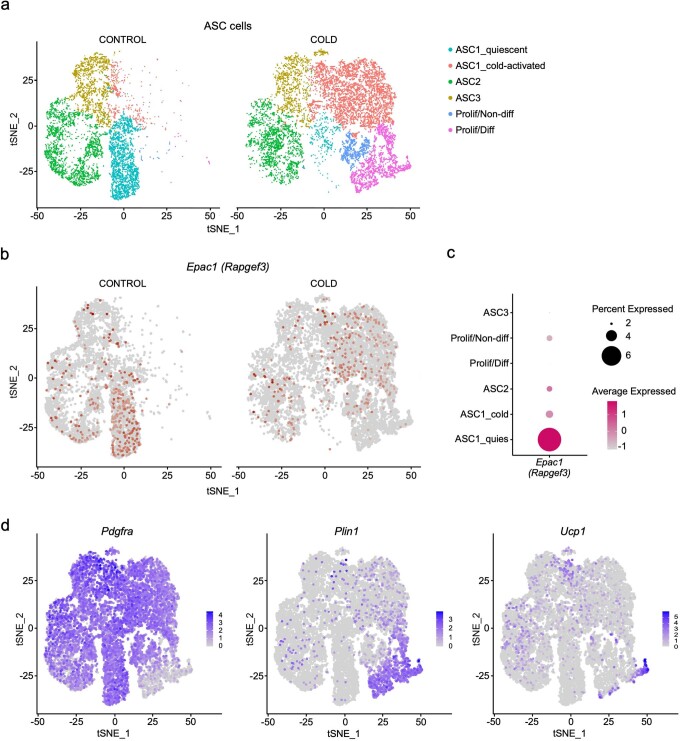
Single-cell RNA sequencing data show high expression of *Epac1* specifically in cold-responsive PDGFRα+ brown preadipocytes. **a**, t-SNE plot generated from published data^28^ (GSE207707) showing the different adipose tissue stromal cells (ASC) and proliferating/differentiating adipocytes in control- and cold-exposed mice. **b**, t-SNE plots from the same dataset showing expression of *Epac1* (*Rapgef3*) in the quiescent ASC1 population (in control mice) and the cold-activated ASC1 population (in cold-exposed mice). **c**, Dot-plot showing the relative expression of *Epac1* (*Rapgef3*) in the different ASC populations. **d**, t-SNE plots showing expression of the preadipocyte marker *Pdgfra* and the mature adipocyte markers *Plin1* and *Ucp1*.

As EPAC1 has an important role in preadipocytes, we focused on PDGFRα^+^ cells, which are precursors for thermogenic adipocytes^27^. Overall, PDGFRα^+^ cells expressed significantly more *Rapgef3* than the PDGFRα-deficient fraction in the BAT (Fig. 3a). Reanalysis of single-cell sequencing data from the BAT of cold-exposed mice^28^ revealed that *Rapgef3* is most highly expressed in a subpopulation of PDGFRα^+^ preadipocytes that is responsive to cold (ASC1) (Extended Data Fig. 5a–d).

Notably, *Pdgfra* expression was lower in the BAT of cold-exposed *Rapgef3*^*−/*^^*−*^ mice compared with cold-exposed WT mice, possibly indicating reduced proliferation of PDGFRα^+^ brown precursor cells in the absence of EPAC1 (Fig. 3b). By contrast, activation of EPAC1 in PDGFRα^+^ cells resulted in significantly increased proliferation compared with in the vehicle-treated cells (Fig. 3c).

**Extended Data Fig. 6:**
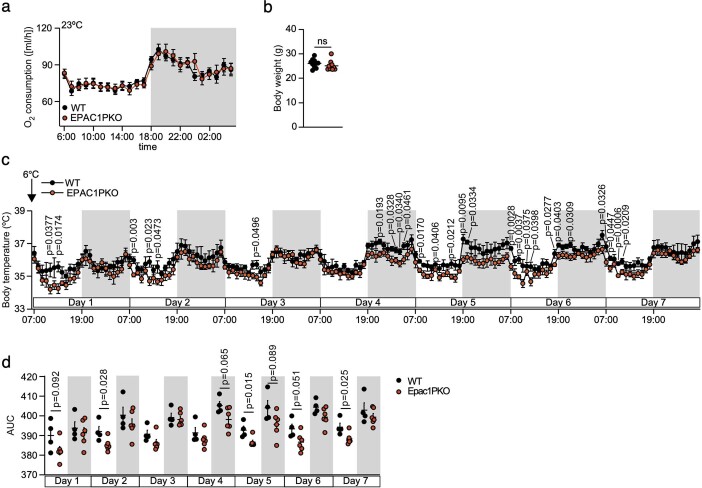
Mice lacking EPAC1 in PDGFRα+ cells. **a**, Oxygen consumption at 23 °C of Epac1^fl/fl^ Pdgfra^Cre/+^ (EPAC1PKO) and Epac1^fl/fl^ Pdgfra^+/+^ (WT) mice over the course of 24 hours, n = 12 (WT), n = 15 (EPAC1PKO) independent animals. **b**, Body weight of mice exposed for 1 week at 4 °C, n = 10 independent animals. **c-d**, Body core temperature was continuously measured using an implanted mouse telemetry system. n = 4 (WT), n = 6 (EPAC1PKO) independent animals. **c**, Body temperature over time; **d**, AUC of day and night cycles were calculated during the cold exposed time. P values determined by unpaired two-tailed t-test. Data are presented as mean values ± SEM. Source numerical data are available in source data. Source data

We therefore generated mice in which *Rapgef3* was selectively knocked out in PDGFRα^+^ cells using *Pdgfra-cre* mice (*Rapgef3*^*fl/fl*^*Pdgfra*^*cre/+*^; hereafter, EPAC1PKO mice). Although EPAC1PKO mice showed no difference in oxygen consumption at room temperature (Extended Data Fig. 6a), EPAC1PKO mice exhibited significantly lower oxygen consumption after cold exposure (Fig. 3d), with no differences in body weight (Extended Data Fig. 6b). Importantly, cold-exposed EPAC1PKO mice displayed significantly decreased BAT mass and significantly decreased UCP1 expression (Fig. 3e–g), mimicking the phenotype of the global-KO mice. Similarly, we observed a decrease in core body temperature, although significant changes between genotypes were observed mainly between day 4 and 7 after cold acclimatization (Extended Data Fig. 6c,d).

Moreover, we crossed *Rapgef3*^*fl/fl*^ with *Ucp1*^*cre/+*^ (*Rapgef3*^*fl/fl*^*Ucp1*^*cre/+*^; hereafter, EPAC1UCP1KO mice) mice. After 1 week of cold exposure, no significant differences in BAT weight, thermogenic markers or oxygen consumption were observed (Fig. 3h–l).

Taken together, EPAC1 in PDGFRα^+^ preadipocytes controls thermogenic adipose tissue mass and ensures adaptive thermogenesis in BAT.

### Loss of EPAC1 in PDGFRα^+^ cells worsens obesity

**Fig. 4:**
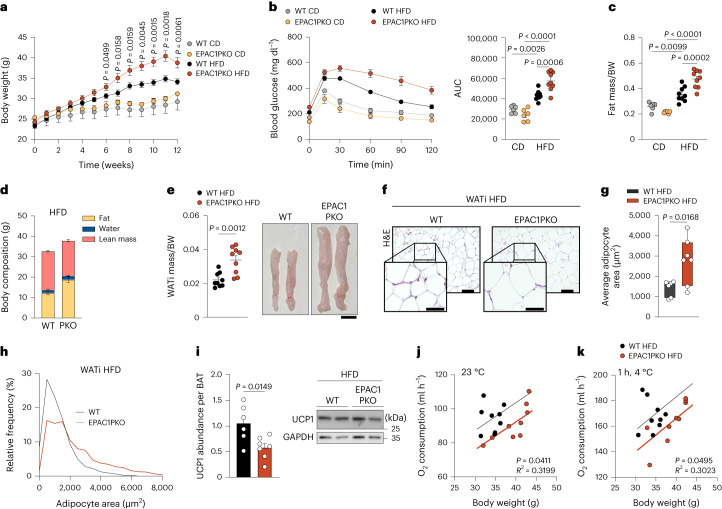
Ablation of EPAC1 in PDGFRα^+^ cells exacerbates the effects of DIO. **a**–**k**, EPAC1PKO and WT (*Rapgef3*^*fl/fl*^*Pdgfra*^*+/+*^) mice were fed an HFD for 12 weeks. **a**, Body weight over the course of 12 weeks. *n* = 9 (HFD) and *n* = 6 (CD) independent animals. **b**, Blood glucose over the course of 120 min (left) and AUC quantification (right). *n* = 9 (HFD) and *n* = 6 (CD) independent animals. **c**, Fat mass per body weight. *n* = 9 independent animals. **d**, The body composition of the HFD-fed groups. *n* = 9 independent animals. **e**, The WATi mass per body weight of HFD-fed mice (*n* = 9 biologically independent samples; left) and representative macroscopy image (right). Scale bar, 1 cm. *n* = 3 biologically independent samples. **f**, Representative H&E immunostaining of WATi after feeding on an HFD. Scale bars, 100 µm (overview) and 50 µm (magnifications). *n* = 3 biologically independent samples. **g**, The average adipocyte area of WATi after feeding on an HFD. *n* = 6 (WT) and *n* = 7 (EPAC1PKO) biologically independent samples. For the box plots, the centre line shows the median, the box limits show the first and third quartiles, and the upper and lower whiskers extend from the maximum to the minimum value. **h**, The relative frequency of adipocyte size of the WATi of HFD-fed mice. *n* = 6 (WT) and *n* = 7 (EPAC1PKO) biologically independent samples. **i**, Representative immunoblot (right) and quantification (left) of UCP1 from BAT after 12 weeks feeding on an HFD. Expression data were normalized to the total BAT weight. *n* = 6 (WT) and *n* = 7 (EPAC1PKO) biologically independent samples. **j**,**k**, Analysis of covariance (ANCOVA) analysis of the oxygen consumption per body weight of mice that were exposed to 23 °C (**j**) and 1 h at 4 °C (**k**) and fed an HFD. *n* = 9 independent animals. *P* values were determined using unpaired two-tailed *t*-tests (**a**, **e**, **g** and **i**) and one-way ANOVA with Tukey’s multiple-comparison test (**b** and **c**). Data are mean ± s.e.m. from biologically independent animals. Source numerical data and unprocessed blots are provided as source data. Source data

**Extended Data Fig. 7:**
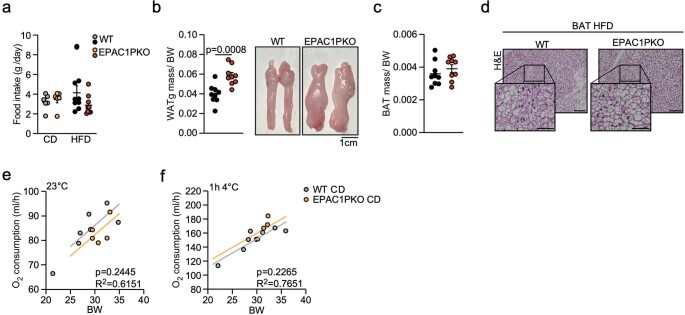
Ablation of EPAC1 in PDGFRα+ cells during diet-induced obesity. Epac1^fl/fl^ Pdgfra^Cre/+^ (EPAC1PKO) and Epac1^fl/fl^ Pdgfra^+/+^ (WT) were fed a HFD for 12 weeks. **a**, Food intake per day in grams n = 9 or a CD, n = 6 independent animals. **b**, WATg mass per body weight of HFD mice, n = 9 biologically independent samples (left panel) and representative macroscopic picture (right panel), n = 3 biologically independent samples. Scale bar 1 cm. **c**, BAT mass per body weight of HFD mice, n = 9 biologically independent samples. **d**, Representative H&E staining of BAT HFD. Scale bars, 100 µm (overview) and 50 µm (magnifications), n = 3 biologically independent samples. **e**, Average oxygen consumption standardized to BW (ANCOVA) of CD mice at 23 °C, n = 6 independent animals. **f**, ANCOVA analysis of oxygen consumption per BW after 1 h 4 °C of mice fed a CD, n = 6 independent animals. P values determined by unpaired two-tailed t-test. In **a**, one-way ANOVA (Tukey’s multiple comparison test). Data are presented as mean values ± SEM. Source numerical data are available in source data. Source data

We next analysed the thermogenic function of EPAC1 in the context of diet-induced obesity (DIO). EPAC1PKO mice fed a high-fat diet (HFD) gained significantly more weight than their WT littermates (Fig. 4a), with no differences in food consumption (Extended Data Fig. 7a). Importantly, EPAC1PKO mice displayed a significantly diminished glucose tolerance (Fig. 4b) and overall increased fat mass (Fig. 4c,d), with significantly higher inguinal WAT (WATi; +53%) and gonadal WAT (WATg; +47%) mass, as well as increased adipocyte size (Fig. 4e–h and Extended Data Fig. 7b). Moreover, UCP1 was significantly decreased in the BAT of the EPAC1PKO mice, although no differences in BAT mass were observed (Fig. 4i and Extended Data Fig. 7c,d). EPAC1PKO mice that were fed an HFD showed a reduced O_2_ consumption rate at 23 °C as well as after 1 h exposure to 4 °C (Fig. 4j,k), whereas no significant differences were observed in the control diet (CD) groups (Extended Data Fig. 7e,f).

Taken together, these data indicate that, under obesogenic conditions, the loss of EPAC1 specifically in PDGFRα^+^ cells reduces the thermogenic capacity of brown/beige fat and exacerbates obesity.

### Activation of EPAC1 counteracts obesity

**Fig. 5:**
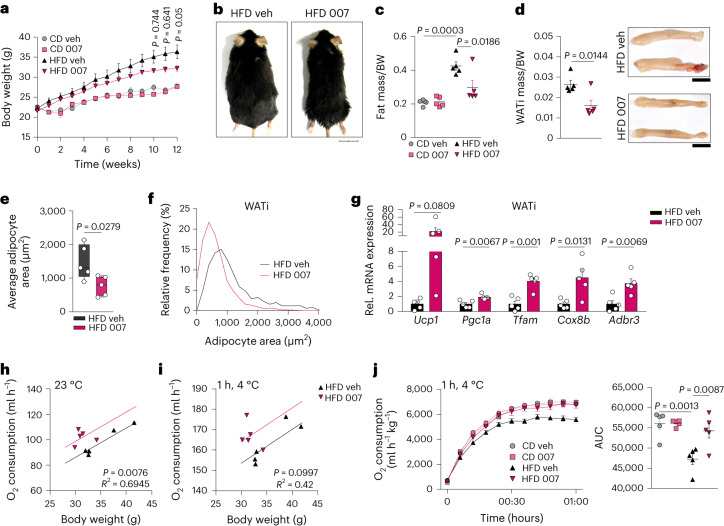
Activation of EPAC1 counteracts obesity. **a**–**j**, Mice were fed a CD or HFD for 12 weeks and injected daily with vehicle (veh) or 007. **a**, The body weight over the course of 12 weeks. *n* = 5 independent animals. **b**, Representative pictures of mice that were injected with vehicle (left) or 007 (right). Scale bar, 2 cm. *n* = 5 independent animals. **c**, Fat mass per body weight. *n* = 5 independent animals. **d**, WATi mass per body weight (left) and representative macroscopy image (right). Scale bars, 1 cm. *n* = 5 biologically independent samples. **e**, The average adipocyte area of WATi after feeding on an HFD. *n* = 5 biologically independent samples. For the box plots, the centre line shows the median, the box limits show the first and third quartiles, and the upper and lower whiskers extend from the maximum to the minimum value. **f**, The relative frequency of adipocyte size of the WATi of HFD-fed mice. *n* = 5 biologically independent samples. **g**, The mRNA expression profile of the WATi of HFD-fed mice. *n* = 5 biologically independent samples. **h**,**i**, ANCOVA analysis of oxygen consumption per body weight of mice that were exposed to 23 °C (**h**) or 4 °C (**i**) and fed an HFD. *n* = 5 independent animals. **j**, Oxygen consumption over the course of 1 h at 4 °C (left) and quantification of the AUC (right). *n* = 5 independent animals. *P* values were determined using unpaired two-tailed *t*-tests (**a**, **d**, **e** and **g**) and one-way ANOVA with Tukey’s multiple-comparison test (**c** and **j**). Data are mean ± s.e.m. from biologically independent animals. Source numerical data are provided as source data. Source data

**Extended Data Fig. 8:**
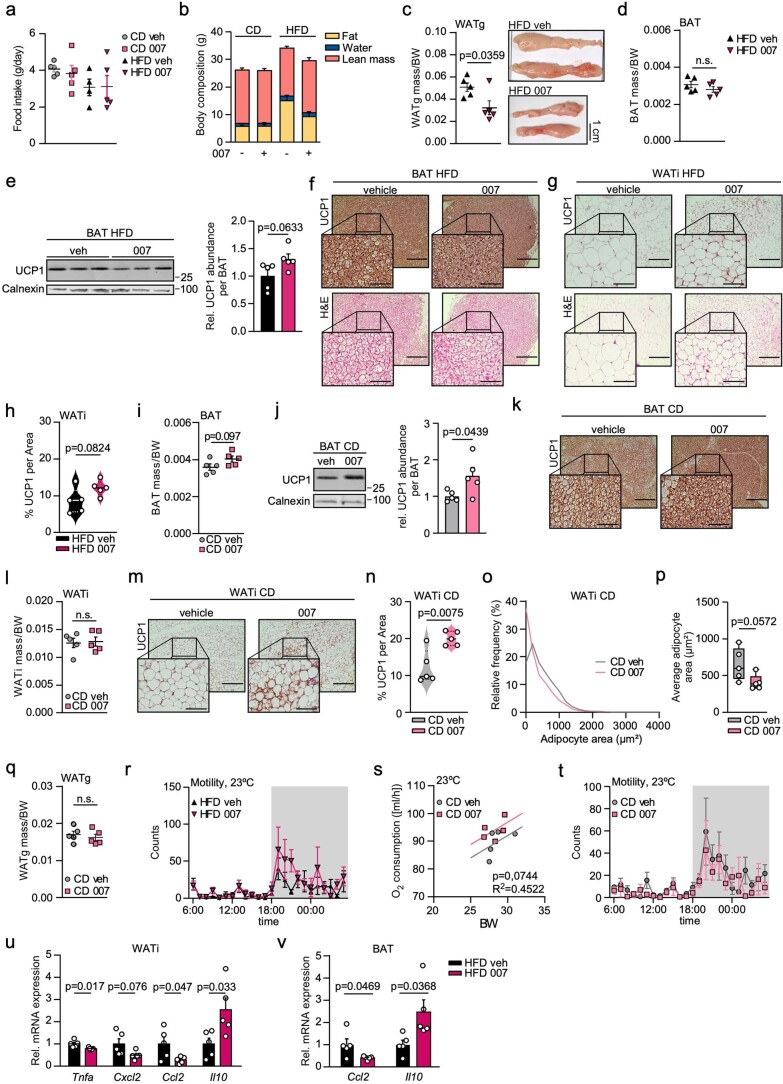
Stimulation of EPAC1 increases EE and browning of WATi and reduces weight gain during diet-induced obesity. **a-v**, Mice were fed a CD or HFD for 12 weeks and injected with NaCl (veh) or 007 as indicated in the figure legends. **a**, Food intake in grams per day, n = 5 independent animals. **b**, Body composition analysis in grams, n = 5 independent animals. **c**, WATg mass per BW of HFD mice, n = 5 biologically independent samples (left panel) and representative macroscopic picture, n = 3 biologically independent samples (right panel). Scale bar 1 cm. **d**, BAT mass per BW of HFD mice, n = 5 biologically independent samples. **e**, Representative immunoblot (left panel) and quantification (right panel) of UCP1 from BAT HFD, expression data normalized to total BAT weight, n = 5 biologically independent samples. **f**, Representative UCP1 (upper panels) and H&E (lower panels) stainings of BAT of HFD mice. Scale bars, 200 µm (overview) and 50 µm (magnifications), n = 3 biologically independent samples. **g**, Representative UCP1 (upper panels) and H&E (lower panels) stainings of WATi of HFD mice, n = 5 biologically independent samples. Scale bars, 200 µm (overview) and 50 µm (magnifications). **h**, Quantification of the percentage of UCP1 positive area, n = 5 biologically independent samples. **i**, BAT mass per BW of CD mice, n = 5 biologically independent samples. **j**, Representative immunoblot (left panel) and quantification (right panel) of UCP1 from BAT CD, expression data normalized to total BAT weight, n = 5 biologically independent samples. **k**, representative UCP1 staining from BAT CD. Scale bars, 200 µm (overview) and 50 µm (magnifications), n = 5 biologically independent samples. **l**, WATi mass per BW of CD mice, n = 5 biologically independent samples. **m-n**, Representative UCP1 staining (**m**) and quantification of the percentage of UCP1 positive area (**n**). Scale bars, 200 µm (overview) and 50 µm (magnifications), n = 5 biologically independent samples. **o**, Relative frequency of adipocyte size in the WATi of CD mice, n = 5 biologically independent samples. **p**, Average adipocyte area of WATi of CD mice, n = 5 biologically independent samples. For the box plots, the centre line shows the median, the box limits show the first and third quartiles, the upper and lower whiskers extend from the maximum to the minimum value. **q**, WATg mass per BW of CD mice, n = 5 biologically independent samples. **r**, Motility of HFD mice over the course of 24 hours, n = 5 independent animals. **s**, ANCOVA analysis of oxygen consumption per BW at 23 °C of CD mice, n = 5 independent animals. **t**, Motility of CD mice over the course of 24 hours, n = 5 independent animals. **u-v**, mRNA profile of (**u**) inflammation markers of WATi and (**v**) inflammation markers of BAT of mice fed a HFD, n = 5 biologically independent samples. P values determined by unpaired two-tailed t-test. In **a**, one-way ANOVA (Tukey’s multiple comparison test). Data are presented as mean values ± SEM. Source numerical data and unprocessed blots are available in source data. Source data

We next studied whether EPAC1 activation could ameliorate obesity. Mice fed an HFD and treated with 007 showed significantly less weight gain (Fig. 5a,b)—with no differences in food intake (Extended Data Fig. 8a)—compared with vehicle-treated HFD-fed mice. The 007-treated HFD-fed mice exhibited a significant decrease in fat mass (Fig. 5c and Extended Data Fig. 8b) with significantly reduced WATi (-39%) and WATg (−36%) mass (Fig. 5d and Extended Data Fig. 8c). No differences in BAT mass were observed; however, the 007-treated HFD-fed group had increased UCP1 levels, and lower lipid accumulation (Extended Data Fig. 8d–f). Treatment with 007 led to a significant decrease in adipocyte area, a higher frequency of smaller adipocytes and an increase in UCP1-positive cells in the WATi (Fig. 5e,f and Extended Data Fig. 8g,h). Notably, the WATi of 007-treated HFD-fed mice also showed 22-fold higher *Ucp1* expression, together with significantly increased levels of *Pgc1a*, *Tfam*, *Cox8b* and *Adbr3* (Fig. 5g). CD-fed mice treated with 007 displayed a tendency for increased BAT mass and significantly higher UCP1 levels (Extended Data Fig. 8i–k). The WATi mass of the 007-treated CD-fed group was not changed, but had a significantly increased number of UCP1-positive cells, as well as smaller adipocytes (Extended Data Fig. 8l–p), indicating increased browning also in the 007-treated CD-fed group. No significant differences in WATg mass were observed in the CD-fed group (Extended Data Fig. 8q).

Whole-body oxygen consumption was significantly increased in 007-treated HFD-fed mice with no differences shown in motility (Fig. 5h and Extended Data Fig. 8r). The maximal cold-induced O_2_ consumption rate in 007-treated HFD-fed mice was increased compared with in the vehicle-treated HFD-fed mice (Fig. 5i), and reached values comparable to those of the CD-fed mice (Fig. 5j). Moreover, EPAC1 activation also increased oxygen consumption (+11%) in mice fed the CD without affecting motility (Extended Data Fig. 8s,t).

The expression of the proinflammatory genes *Tnf* and *Ccl2* was decreased in the WATi of 007-treated HFD-fed mice, whereas the anti-inflammatory marker *Il10* was upregulated (Extended Data Fig. 8u). Moreover, the BAT from 007-treated HFD-fed mice exhibited lower levels of *Ccl2* and increased levels of *Il10* (Extended Data Fig. 8v).

Overall, these results demonstrate that EPAC1 signalling counteracts obesity-induced metabolic alterations, reduces adipose tissue inflammation and increases the function of thermogenic fat during feeding on an HFD.

### Role of EPAC1 in human brown adipocytes

**Fig. 6:**
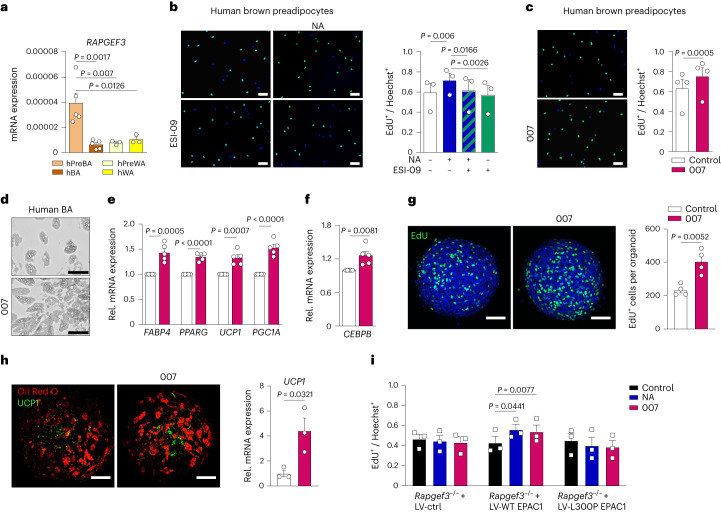
Activation of EPAC1 induces human brown adipocyte proliferation and differentiation. **a**, *RAPGEF3* mRNA expression ($2{^{-\Delta C{_{\mathrm{t}}}}}$) in human brown versus white preadipocytes (hPreBA and hPreWA, respectively) and mature brown and white adipocytes (hBA and hWA). *n* = 5 (human brown adipocytes) and *n* = 3 (human white adipocytes) independent experiments. **b**,**c**, Representative EdU staining (left; green, EdU; blue, DAPI) and quantification (right) of proliferating cells that were pretreated with or without ESI-09 and stimulated with or without NA (**b**; *n* = 3 biologically independent samples) or stimulated with or without 007 (**c**; *n* = 4 biologically independent samples). For **b** and **c**, scale bars, 100 µm. **d**, Representative images of human brown adipocytes (hBAs) that were differentiated with or without 007. Scale bars, 100 µm. *n* = 3 biologically independent samples. **e**, Relative mRNA expression of *FABP4*, *PPARG*, *UCP1* and *PGC1A* of human brown adipocytes that were differentiated with or without 007. *n* = 5 biologically independent samples. **f**, Relative *CEBPB* expression 8 h after induction with or without 007. *n* = 6 biologically independent samples. **g**, Analysis of proliferation in hiPSC-BAP organoids that were stimulated with or without 007 and labelled with EdU. Representative images (left) and quantification (right). *n* = 4 independent experiments. Scale bars, 100 µm. **h**, hiPSC-BAP organoids were differentiated with or without 007. Representative images of Oil Red O (red) and UCP1 (green) staining (left) are shown. Scale bars, 100 µm. Right, relative *UCP1* expression. *n* = 3 independent experiments. **i**, Quantification of proliferating primary *Rapgef3*^*−/−*^ brown preadipocytes that were transduced with lentiviruses expressing IRES-GFP (LV-ctrl), WT EPAC1 (LV-WT EPAC1) or EPAC1(L300P) mutant (LV-L300P EPAC1), and treated with or without vehicle, NA or 007. *n* = 3 independent experiments. *P* values were determined using unpaired two-tailed *t*-tests (**c** and **e**–**h**), and one-way (**a**), one-way repeated-measures (**b**) and two-way repeated-measures (**i**) ANOVA with Tukey’s multiple-comparison test. Data are mean ± s.e.m. Source numerical data are provided as source data. Source data

**Extended Data Fig. 9:**
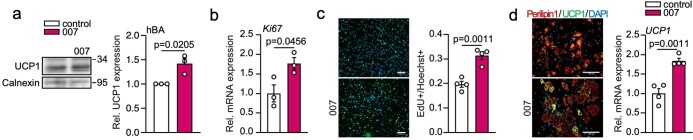
Activation of EPAC1 induces hiPSC-BAP proliferation and differentiation. **a**, Representative immunoblot (left panel) and quantification (right panel) of UCP1 in hBA treated with or without 007, n = 3, biologically independent samples. **b**, Relative *Ki67* expression of human induced pluripotent stem cells derived brown-like adipose progenitors (hiPSC-BAP) organoids stimulated with or without 007, n = 3 independent experiments. **c**, hiPSC-BAPs stimulated with or without 007. Proliferating cells labelled with EdU (green). DNA (blue) stained with DAPI. Green cells show EdU/DAPI-positive cells, n = 4 independent experiments. **d**, hiPSC-BAPs differentiated in the presence or absence of 007 from d-1 to d24, representative images of Perilipin1 (red), UCP1 (green) and DAPI (blue) staining (left panel) and quantification of relative *UCP1* expression (right panel), n = 4 independent experiments. P values determined by unpaired two-tailed t-test. Data are presented as mean values ± SEM. Source numerical data and unprocessed blots are available in source data. Source data

*RAPGEF3* expression was significantly increased in human brown preadipocytes compared with in mature human brown adipocytes and human white adipocytes (Fig. 6a). Human brown preadipocytes exhibited increased proliferation after stimulation with NA, which was blunted after concomitant treatment with the EPAC inhibitor ESI-09 (Fig. 6b). By contrast, we observed a significant increase in proliferation after stimulation with 007 (Fig. 6c). Moreover, human brown adipocytes that were treated with 007 had increased lipid-droplet accumulation and significantly increased expression of *FABP4* and *PPARG*, as well as *PGC1A* and *UCP1* (protein and mRNA) (Fig. 6d,e and Extended Data Fig. 9a). Similar to mouse adipocytes, treatment with the EPAC1 activator increased the expression of *CEBPB* (Fig. 6f).

Next, human induced pluripotent stem cells (hiPSCs) were used to generate human beige/brown-like adipose progenitors (hiPSC-BAPs)^29^ for 3D cultures. Importantly, the 007-treated hiPSC-BAP organoids showed a significant increase in EdU-positive cells, alongside an increase in the proliferation marker *MKI67* (encoding Ki-67; Fig. 6g and Extended Data Fig. 9b). At the end of differentiation, hiPSC-BAP organoids that were stimulated with 007 had increased *UCP1* mRNA levels (Fig. 6h). Moreover, when cultured in 2D, hiPSC-BAPs treated with 007 showed an increase in proliferation in preadipocytes, combined with an increase in *UCP1* expression (Extended Data Fig. 9c,d). Together, these data indicate that EPAC1 activation induces the proliferation and differentiation of human brown adipocytes as well as of human iPSC-derived brown adipocytes in 3D/organoids, which might have relevance also for translational approaches directed at transplantation of human BAT precursors to increase thermogenic fat mass.

Notably, the low-frequency coding variant p.Leu300Pro (rs145878042) in *RAPGEF3* was shown to positively correlate with BMI in a cohort of 718,734 individuals^30^. Studies of the effect of this Pro300 substitution on EPAC1 function are lacking. To investigate whether this mutation leads to a loss of the pro-proliferative effect of EPAC1 in preadipocytes, we transduced murine *Rapgef3*^*−/−*^ preadipocytes and reconstituted them with either WT EPAC1 or the EPAC1 variant (EPAC1(L300P)) using lentiviral vectors. EdU-incorporation experiments revealed that preadipocytes that were reconstituted with WT EPAC1 showed increased proliferation in response to either NA or 007. By contrast, neither 007 nor NA affected proliferation in preadipocytes transduced with EPAC1(L300P) or an empty control vector (Fig. 6i). These data strongly suggest that the Leu300Pro has a negative effect on BAT mass and energy homeostasis; thereby leading to increased BMI in humans carrying this variant.

## Discussion

BAT activity decreases during aging and in obesity^4,31^. Thus, deciphering the mechanisms behind BAT growth is essential to enhance energy expenditure in older patients or patients with obesity. The main focus of BAT research has been on the cAMP–PKA signalling pathway; however, it has also been described that noradrenergic stimulation can induce proliferation of brown preadipocytes through ERK1/2 in a cAMP/PKA-independent manner^32^. Although several studies investigated the role of EPAC proteins in metabolism, the role of EPAC1 in thermogenic fat and energy homeostasis is not clear. As previous studies showed that EPAC activation was insufficient to induce differentiation of white adipocytes, a role of EPAC1 in thermogenic adipocyte differentiation was not expected^33^.

Moreover, previous studies used different genetic mouse models resulting in partially conflicting results^13,14^. One study^14^ generated *Rapgef3*-KO mice by replacing exons 8 to 21 of the *Rapgef3* gene with a neomycin cassette. These KO mice have defects in pancreatic β-cell insulin secretion and more severe DIO. By contrast, using a different *Rapgef3*-KO mouse model, a study found^13^ that ablation of EPAC1 resulted in reduced obesity, which was explained by enhanced leptin sensitivity in the brain. However, Yan et al.^13^ replaced exons 3–5, affecting only the DEP domain of the regulatory region.

Here we used a multipronged approach to decipher the role of EPAC1 in thermogenic adipose tissue and energy homeostasis: (1) we used a global *Rapgef3*-KO model based on the insertion of *loxP* sequences within introns 7 and 15 of the *Rapgef3* gene^11,34^. Detailed metabolic analysis of these mice revealed impaired BAT growth and reduced thermogenic ability after physiological BAT activation. (2) Pharmacological activation of EPAC1 with 007 increased brown fat mass and energy expenditure. These effects of 007 were absent in EPAC1-deficient adipocytes and *Rapgef3-*KO mice, demonstrating the specificity of this EPAC1 activator. (3) To pinpoint the function of EPAC1, we used a cell-specific KO mouse model.

EPAC1 is highly expressed in PDGFRα^+^ adipocyte progenitor cells present in the stromal vascular fraction (SVF) of BAT. Furthermore, analysis of single-cell sequencing data^28^ revealed that a subpopulation of PDGFRα^+^ cells that is cold-responsive expresses the highest level of EPAC1. Previous studies showed that PDGFRα^+^ cells give rise to brown adipocytes and that beige cells in WAT are derived from SVF cells that express PDGFRα at high levels^35,36^. The loss of EPAC1 in PDGFRα^+^ preadipocytes resulted in a decrease in oxygen consumption and BAT mass as well as worsened DIO. Our data clearly show that EPAC1 is a major regulator of energy expenditure through the regulation of the number of thermogenic adipocytes.

Importantly, the positive effect of EPAC1 on differentiation is specific for thermogenic adipocytes and was not observed in white preadipocytes/during white adipogenesis. In this context, it is interesting that adrenergic stimulation promotes brown, but inhibits white, preadipocyte proliferation^37^. As white adipocyte proliferation is a hallmark of obesity^38^, selectively enhancing proliferation of thermogenic adipocytes may be an especially valuable approach in patients with obesity.

To elucidate the molecular mechanisms that are responsible for the EPAC1 effects, we first performed phosphoproteomic analysis. This revealed that EPAC1 specifically regulates the mTORC1 pathway, which is crucial for anabolic growth during cell proliferation. Moreover, we found that EPAC1 activation increases phosphorylation of ERK1/2, an important initiator and regulator of mitosis. Functional validation of these findings demonstrated that (1) mTORC1 and ERK1/2 activity is essential for EPAC1-induced preadipocyte proliferation, and (2) EPAC1 activates ERK1/2 and stimulates proliferation exclusively in brown but not white preadipocytes. Notably, we found that EPAC1 is required for NA-induced brown preadipocyte proliferation, indicating a central role of EPAC1 in cold-induced growth of BAT. Furthermore, we found an increased expression of C/EBPβ—a central transcription factor for brown but not white adipocyte differentiation—after EPAC1 activation.

Regarding the role of EPAC1 in mature adipocytes, EPAC1 has previously been shown to regulate glucose uptake through mTORC2 signalling in differentiated brown adipocytes^39^. Overall, this EPAC1/mTORC2-induced increase in nutrient uptake could potentially support the anabolic response associated with NA/EPAC1/mTORC1-mediated BAT growth. Moreover, it was recently reported that EPAC proteins are involved in acute downregulation of β_3_-adrenergic receptors in differentiated 3T3-L1 cells and visceral white fat^40^. In contrast, we found that activation of EPAC1 during feeding on an HFD leads to an increase in β_3_-adrenergic receptors in subcutaneous WAT.

The finding that pharmacological activation of EPAC1 enhanced human brown adipocyte proliferation and differentiation in both conventional 2D cultures as well as in 3D/organoid models demonstrates the importance of EPAC1 for human thermogenic adipocytes and energy homeostasis. Apart from pharmacological activation of proliferation and differentiation of resident thermogenic fat cells, transplantation of brown/beige precursors, which can be derived from iPSCs, has been put forward as an alternative strategy to increase the number of thermogenic adipocytes in humans^41^. Thus, one can envision pretreating iPSC-derived brown/beige precursor cells with an EPAC1 activator to improve the efficacy of such brown/beige fat transplantation approaches.

Finally, we identified a single-nucleotide polymorphism (p.Leu300Pro) of *RAPGEF3*, which has recently been shown to positively correlate with BMI in a cohort of 718,734 individuals^30^, as a missense mutation that interferes with NA-induced brown adipocyte proliferation.

Taken together, our data identify EPAC1 as a major regulator of brown/beige adipocyte proliferation and differentiation. EPAC1 might be a potential pharmacological target to promote human brown/beige adipose tissue growth, energy expenditure and cardiometabolic health that could be directly targeted using pharmacological activators. Alternatively, ex vivo activation of EPAC1 in iPSC-derived autologous human brown/beige preadipocytes might be a way to enhance transplantation-based approaches to increase BAT mass and, consequently, energy expenditure and/or the release of protective endocrine factors (BATokines)^42^ to combat diabetes and obesity.

## Methods

All of the animal studies were performed in accordance with national guidelines and were approved by the Landesamt für Natur, Umwelt und Verbraucherschutz, Nordrhein-Westfalen, Germany (84-02.04.2017.A314; 81-02.04.2019.A254) and by the Behörde für Gesundheit und Verbraucherschutz Hamburg, Germany (N082/2020).

### Animal studies

WT male C57Bl/6J mice were purchased from Charles River.

*Rapgef3*^*−/−*^ (ref. ^34^) mice and *Rapgef3*^*fl/fl*^ mice were provided by F. Lezoualc’h. *Pdgfra-cre* ((C57BL/6-Tg(*Pdgfra-cre*)1Clc/J, 013148) and *Ucp1-cre* (B6.FVB-Tg(*Ucp1-cre*)1Evdr/J, 024670) mice were purchased from Jackson. Only male mice were used.

All mice were maintained on a daily cycle of 12 h light (06:00–18:00) and 12 h darkness (18:00–06:00), at ambient room temperature (23 ± 1 °C) and 40–50% humidity if not otherwise stated, and were allowed free access to chow and water.

Sample sizes for all in vivo experiments are given in the figure legends.

#### HFD

Male mice (aged 7 weeks) were fed with an HFD (Ssniff, D12492) or a CD (Ssniff, D12450B) for 12 weeks and the body weight was monitored weekly.

#### Energy expenditure

Oxygen consumption was measured using the PhenoMaster (TSE Systems) system for 120 s per mouse every 18 min for 24 h. During the study, mice were maintained on a daily cycle of 12 h light (06:00–18:00) and 12 h darkness (18:00–06:00). For long-term cold exposure measurements, 7-week-old mice were housed at 16 °C for 3 days and kept at 4 °C for a week. For thermoneutrality experiments, 7-week-old mice were housed at 30 °C for a week.

#### Body temperature

To measure body temperature, transponders (G2 E-Mitter) were transplanted into the peritoneum of anaesthetized eight-week-old male mice. Then, 7 days after surgery, the recovered mice were single caged in a thermally and humidity-controlled environment using the Promethion system (Sable Systems). Body core temperatures were recorded at 6 °C as indicated in the figure legend.

#### Pharmacological activation of EPAC

For the short-term studies, 8-week-old male C57Bl/6J WT mice were injected with NaCl or 007 (2 mg per kg body weight) intraperitoneally for 1 week before measurement. For the DIO studies, 7-week-old C57Bl/6J WT male mice were used at the start of the experiment. Mice were injected intraperitoneally once daily with 007 (2 mg per kg) or with NaCl for 12 weeks.

Oxygen consumption was measured every 18 min for 120 s during 24 h with Phenomaster (TSE Systems). For short-term cold exposure (1 h 4 °C), the mice were measured for 120 s every 6 min for 1 h.

#### Body composition analysis

Body composition was analysed using a table Bruker Minispec LF50H system.

#### Glucose-tolerance test

Animals were fasted for 5 h. 8 µl per g body weight of glucose solution (0.25 g ml^−1^) was injected intraperitoneally and glucose was measured at the indicated timepoints after injection. The tail vein was punctured and blood was analysed using the Accu Check (Aviva Nano) analyzer and dipsticks (Roche).

### Brown adipocyte isolation and differentiation

BAT-derived mesenchymal stem cells were isolated from interscapular BAT of 4–5 newborn WT mice and isolated as previously described^43^. Preadipocytes were immortalized using the lentivirus containing the SV40 large T antigen and unselected cells were cultured in DMEM supplemented with FBS and penicillin–streptomycin (GM). The cells were expanded in GM at 37 °C under 5% CO_2_.

For differentiation, immortalized cells were plated at a density of 167,000 cells per well in a six-well plate in GM. Then, 48 h after seeding (day −2), GM was replaced by differentiation medium (DM), supplemented with FBS, penicillin–streptomycin, 20 nM insulin and 1 nM triiodothyronine. Confluent cells (day 0) were treated for 48 h with BAT induction medium (DM supplemented with 0.5 mM isobutylmethylxanthine (IBMX) and 1 μM dexamethasone). After induction, cells were treated with DM for the next 5 days, changing the medium every other day. Where indicated, cells were treated every second day with 007 (Biolog, C 041). In the experiments in which U0126 (Tocris, 1144) and ESI-09 (Tocris, 4773) were used, inhibitors were added 15 min before the addition of 007 or NA (Sigma-Aldrich, A9512).

For preadipocyte experiments on ERK1/2 phosphorylation and C/EBPβ expression, cells were used on day −2 and on day 0, respectively. For mature brown adipocyte experiments, cells were used on day 7 after induction.

Primary brown adipocytes (non-immortalized) were used for the study of proliferation (EdU assays) and to assess the differences between WT and *Rapgef3*^*−/*^^*−*^ brown adipocytes.

### Lentiviral constructs

Third-generation lentiviral particles were produced by calcium-phosphate-based transfection of HEK293T cells. Viral particles were concentrated by ultracentrifugation. CRISPR–Cas9 experiments were performed using the lentiCRISPR V2 plasmid system with gRNAs targeting *Cebpb*. lentiCRISPR v2 was a gift from F. Zhang (Addgene plasmid, 52961)^44^. Lentiviral doses corresponding to 200 ng of viral reverse transcriptase per well were used for each experiment.

### White adipocyte isolation and differentiation

Primary white adipocytes were isolated from 8–12-week-old C57BL/6 mice and were used to perform the experiments.

Dissected WATi from 2–3 different mice was digested in DMEM (Invitrogen) containing 0.5% BSA and collagenase type II at 37 °C and then centrifuged at 250*g* for 10 min. The resulting pellet was resuspended and filtered using a 100 µm nylon mesh. The filtered solution was seeded in a T175 culture flask in DMEM supplemented with 10% FBS and 1% penicillin–streptomycin (WA growth medium) and kept at 37 °C under 5% CO_2_. Then, 24 h after seeding, cells were washed with PBS and maintained with WA growth medium at 37 °C, 5% CO_2_. The medium was refreshed every other day until cells reached confluency and were subsequently cryopreserved.

Cells were plated at a density of 140,000 cells per well in a six-well plate in growth medium. Cells were grown to confluence in growth medium. Once confluent, preadipocytes were induced during 48 h (day 0 to day 2) by changing the medium to WA induction medium (DMEM containing 5% FBS, 1% penicillin–streptomycin, 1 nM T3, 0.172 µM insulin, 50 mg ml^−1^ l-ascorbate, 1 mM d-biotin, 17 mM pantothenate, 1 µM rosiglitazone, 0.25 µM dexamethasone and 0.5 mM 3-isobutyl-1-methylxantine). From day 2 until day 12, cells were maintained in WA maintenance medium (DMEM containing 5% FBS, 1% penicillin–streptomycin, 1 nM T3, 0.172 µM insulin, 50 mg ml^−1^ l-ascorbate, 1 mM d-biotin, 17 mM pantothenate), refreshing it every other day. Starting on day 0, cells were chronically treated with 007 (Biolog, C 041) as stated in the figure legends. Medium and treatment were refreshed every other day. Cells were analysed on day 12. For all preadipocyte experiments, cells were used on day 0.

To obtain beige adipocytes, primary white adipocytes were treated with 1 µM NA throughout differentiation (day 0 to day 12). UCP1 was assessed at the end of differentiation to ensure that beige cells of WATi origin were obtained.

### Differentiation of human brown adipocytes

Primary human brown adipocytes were cultured as described previously^45^. Starting on the day that cells were confluent, cells were chronically treated with 007 and analysed on day +12. For the analysis of *CEBPB*, cells were induced on day 0 for 8 h and concomitantly treated with 007.

### Generation of hiPSC-BAPs

To generate organoids, a suspension of hiPSC-BAPs at 1 × 10^6^ cells per ml was prepared in growth medium (DMEM enriched with FBS, 2.5 ng ml^−1^ FGF2, penicillin–streptomycin, l-glutamine and HEPES). Then, 5 × 10^4^ cells were seeded in each well of 96-well ultra-low-attachment plate (Corning, 7007). Organoids were generated overnight (day −1). To differentiate the organoids (day 0), organoids were incubated with differentiation medium: endothelial cell basal medium 2 (Promocell, C-22211) supplemented with 0.1% FCS, IBMX (0.5 mM), dexamethasone (0.25 μM), T3 (0.2 nM), insulin (170 nM), rosiglitazone (1 μM), SB431542 (5 μM) and EGM-2 cocktail (Lonza, CC-3121) including ascorbic acid, hydrocortisone and EGF. On day 3, cells were fed differentiation medium without IBMX and dexamethasone. Insulin, T3 and FCS were still maintained in differentiation medium after day 9. Organoids were maintained in differentiation medium until day 24, with the medium changed every 3 days.

For labelling with EdU, organoids were incubated with EdU overnight in the presence or absence of 007 from day −1 to day 3. EdU detection and DAPI staining was performed according to the manufacturer’s instructions (Invitrogen, C10337)^46^.

For the experiments described in Fig. 6h, organoids were incubated with 007 from day −1 to day 24; medium and treatment were refreshed every 3 days.

### RNA isolation, cDNA reverse transcription and qPCR

Isolation of RNA from cells/tissue was performed using the innuSOLV RNA Reagent (Analytik Jena, 845-SB-2090100) according to the manufacturer’s instructions. The final concentration of RNA was quantified using the Nanodrop Spectrophotometer. According to the manufacturer’s instructions, 500–1,000 ng of RNA was transcribed using the Transcriptor First Strand cDNA Synthesis Kit (Roche, 4896866).

mRNA expression of the target genes was amplified and quantified using the transcribed cDNA. mRNA expression was assessed using qPCR using the HT7900 instrument from Applied Biosystems and SYBR-Green PCR master mix (Applied Biosystems, 4309155). Quantification of mRNA levels was performed on the basis of the crossing-point values of the amplification curves using the second derivative maximum method. *Hprt* was used as an internal control for the mouse samples and *GAPDH* was used for the human samples.

The primer sequences are shown in Supplementary Table 1.

### Western blotting and quantification

Cells/tissues were homogenized in RIPA buffer containing protease and phosphatase inhibitors. After centrifugation (10,000*g*, 30 min, 4 °C), the supernatants were collected, and the protein concentration was determined using the Bradford reagent. Western blot analysis was performed according to the manufacturer’s instructions. All antibodies were used at a 1:1,000 dilution. The following antibodies were used: UCP1 (D9D6X) (Cell Signaling, 14670S), FABP4 (Cell Signaling, 2120S), PPARγ (D69) (Cell Signaling, 2430S), EPAC1 (5D3) (Cell Signaling, 4155), phosphoERK1/2 (Thr202/Tyr204) (Cell Signaling, 9101), ERK1/2 (Cell Signaling, 9102), C/EBPβ (Santa Cruz, sc-150), GAPDH (14C10) (Cell Signaling, 2118) and calnexin (EMD Millipore, 208880, 3517587). The bands were visualized using the Odyssey imaging system (LI-COR Bioscience) with fluorescence-labelled secondary antibodies (anti-rabbit IgG (H+L), Dylight 800, 4× PEG conjugate, 5151; anti-mouse IgG (H+L)-DyLight 800 4× PEG conjugate, 5257, Cell Signaling Technology) according to the manufacturer’s protocol.

### In vitro/ex vivo oxygen consumption and UCP1-dependent respiration

For in vitro analyses, brown adipocytes were treated as described above and used at day 7.

For ex vivo analyses, mice were injected during 7 days with 2 mg per kg of 007 or with NaCl. A total of 5–6 mg BAT was dissected and incubated with or without 1 µM NA for 30 min.

Experiments were conducted using the Oxygraph 2K (Oroboros Instruments) system. Samples (cells/tissues) were transferred to the Oxygraph chamber containing 2 ml incubation medium (0.5 mM EGTA, 3 mM MgCl_2_ 6H_2_O, 60 mM K-lactobionate, 20 mM taurine, 10 mM KH_2_PO_4_, 20 mM HEPES, 110 mM sucrose and 1 g l^−1^ BSA, pH 7.1). In vitro/ex vivo respiration levels were recorded when reaching a steady state followed by addition of substrates (1, endogenous; 2, digitonin; 3, pyruvate-malate-glutamate followed by succinate; 4, GDP; 5, FCCP). Respiration rates were normalized to total protein content or to mg of tissue and UCP1 respiration was calculated from the difference between GDP and succinate respiration rates.

### Determination of lipid-droplet size

Adipocytes were treated as indicated in the figure legends. On the last day of differentiation, images were acquired through automated digital microscopy using the Cytation 5 (BioTek) system. Three images per well were automatically taken. Image processing and calculation of the average lipid-droplet size as well as of the lipid-droplet-covered area was performed using the Gen5 software provided (minimum object size: 1 µm; maximum object size: 40 µm).

### LipidTOX staining

Brown preadipocytes were seeded onto clean 18 mm round glass coverslips and differentiated into mature adipocytes (day 7). Cells were washed twice with PBS for 5 min each and fixed with 4% PFA in PBS (pH 7.4) for 10 min at room temperature. Mature cells were washed four times with PBS for 5 min each. Subsequently, the samples were incubated with lipidTOX (H34475, Thermo Fisher Scientific) for 30 min at room temperature. Cells were rinsed with PBS then incubated with RedDot2 (40061, Biotium) for 30 min at room temperature. The samples were washed four times with PBS for 5 min each and kept in PBS until imaging.

### PDGFRα^+^ cell isolation

To isolate PDGFRα^+^ cells from BAT, magnetic-activated cell sorting (MACS) was used. For the best yield of PDGFRα^+^ cells, we pooled the BAT from five to six 8–12-week-old WT C57Bl/6J mice. Tissue was minced and digested in digestion buffer (DMEM containing 0.5% BSA and 1.5 mg ml^−1^ collagenase II). After digestion, all tissue debris was removed by filtration using a 100 µm nylon mesh (Merck Milipore, NY1H00010). PDGFRα^+^ cells were then isolated using the CD140a (PDGFRα) MicroBead Kit, mouse (Miltenyi Biotec, 130-101-502) and liquid separation columns (Miltenyi Biotec, 130-042-401) according to the manufacturer’s instructions.

### Phosphoproteomics

PDGFRα^+^ cells from BAT were isolated and plated until 80% confluence. Cells were starved for 1 h before stimulation with 007 or 6-MB for 30 min. Phosphopeptides were enriched starting with 1 mg of protein input using the Easyphos workflow^47^ and analysed using mass spectrometry (MS) on the Thermo Orbitrap Exploris 480 mass spectrometer. Phopshopeptides were loaded onto a 50 cm column, maintained at 60 °C, with a 75 µM inner diameter. This column was packed in-house with 1.9 µM C18 ReproSil particles from Dr. Maisch. The separation of peptides was achieved through a 120 min gradient by reversed-phase chromatography with a binary buffer system comprising 0.1% formic acid (buffer A) and 80% acetonitrile (ACN) in 0.1% formic acid (buffer B). The acquisition followed a data-dependent cycle with a 1 s time interval, a maximum injection time of 80 ms, a scan range of 300–1,650 Th and an AGC target of 300%, without the use of FAIMS. Sequencing of peptides was achieved through higher-energy collisional dissociation fragmentation with a target value of 1 × 10^5^ and a window of 1.4 Th. Survey scans were performed at a resolution of 60,000 and, for HCD spectra, the resolution was set to 15,000, with a maximum ion-injection time of 50 ms. Dynamic exclusion was configured for 40 s, and an apex trigger was enabled. The acquired raw MS data were processed using MaxQuant v.2.0.1.0, with the ‘match between runs’ feature enabled. The setting for ‘max. missed cleavages’ was set to 2, and the default settings were used unless otherwise specified. ANOVA and two-sided unpaired Student’s *t*-tests were used to filter significantly changed phosphopeptides after width adjustment to correct for protein amount and imputation of missing values by random numbers drawn from a normal distribution with a width of 0.3 and down shift of 1.8 using Perseus. For unsupervised hierarchical clusterings, *z*-scored intensity values were used.

The MS proteomics data have been deposited at the ProteomeXchange Consortium via the PRIDE partner repository under dataset identifier PXD046709.

### EdU labelling

Primary brown preadipocytes (non-immortalized) were seeded onto coverslips at a density of 20,000 cells per well in 24-well plates. Primary white preadipocytes and primary human brown preadipocytes were seeded at a density of 10,000 cells per well. Then, 16 h after seeding, cells were treated as indicated in the figure legends and EdU was added. ESI-09, U0126 and rapamycin were added 15 min before NA or 007. Cells were fixed 48 h after and stained according to the manufacturer’s instructions (Invitrogen, C10337).

For Fig. 6i, *Rapgef3* cDNA was isolated from reverse-transcribed BAT mRNA by PCR amplification and inserted into the lentiviral vector 156rrlsinPPTCMV (provided by L. Naldini) followed by an internal ribosomal entry site (IRES) and eGFP cassette. The L300P (T899C) mutation was inserted by mutagenesis PCR using Q5 Hot start High-Fidelity DNA Polymerase (New England Biolabs). For EdU experiments in *Rapgef3*^*−/−*^ preadipocytes, BAT SVF was isolated from 8–12-week-old *Rapgef3*^*−/*^^*−*^ mice, seeded onto glass coverslips at a density of 10,000 cells per cm^2^, and transduced 8 h later with the indicated lentiviral vectors at a multiplicity of infection of 100. Then, 48 h after seeding, the cells were treated with EdU (10 µM) together with either 200 µM 8-pCPT-2-*O*-Me-cAMP-AM (Biolog) or 1 µM NA. The cells were allowed to proliferate for a further 48 h, after which they were fixed with 4% PFA and assayed using the Click-iT Plus EdU Imaging Kit (C10638, Thermo Fisher Scientific) according to the manufacturer’s instructions.

### DAPI staining

Cells were differentiated as described, fixed with PBS containing 4% PFA and stained with 1 µg ml^−1^ DAPI. Images were processed in MIPAR image processor (SciSpot Scientific Solutions). Cell counts were estimated using the ‘Count Colonies 2’ recipe using the default settings.

### Oil Red O staining

Differentiated adipocytes were washed twice with PBS, fixed with 4% paraformaldehyde at room temperature for 15 min and washed twice again with PBS. Cells were then stained with 5 mg ml^−1^ Oil red O in isopropanol (O0625, Sigma-Aldrich) at room temperature for 2 h. Next, cells were washed three times with tap water and left to dry at room temperature. For visualizing, the Epson Perfection V370 Photo scanner was used.

### Lipolysis

Cells were incubated with 300 µl of lipolysis medium (2% BSA in DMEM) at 37 °C, 5% CO_2_ for 2 h. After incubation, the supernatant was incubated with free glycerol reagent (Sigma-Aldrich, F6428) for 5 min at 37 °C and absorption was measured at 540 nm and 600 nm as reference wavelength using a plate reader (PerkinElmer, Enspire).

### IHC analysis

BAT, WATi and WATg were fixed in PBS containing 4% PFA for 20 h and dehydrated using ethanol. The paraffin-embedded tissue was cut into 5 μM sections.

For UCP1, histological sections were blocked with 2% goat serum-TBS (tris-buffered saline) for 30 min (at room temperature). Immunohistochemistry (IHC) stainings were performed using a primary UCP1 antibody (1:75) (custom made, Thermo Fisher Scientific) overnight. Incubation with a secondary antibody conjugated with horseradish peroxidase (SignalStain Boost IHC; Cell Signaling, 8114S) was performed for 1 h at room temperature. Sections were visualized using 3,3′-diaminobenzidine (DAB) substrate (Vector Laboratories).

For H&E staining, the sections were stained with haematoxylin (Merck, 1.09249) for 2 min and rapidly washed with distilled water. Staining with eosin (Merck, 1.09844) was performed immediately after by incubating the tissue section in eosin for 1 min and then washing for 4 min with distilled water.

The average adipocyte size and the adipocyte frequency was calculated as described previously^48^. UCP1 DAB staining was quantified using ImageJ as described previously^49^.

### Statistics and reproducibility

To determine the group size necessary for sufficient statistical power, power analysis was performed using PS Power and Sample Size Calculation software using preliminary data, and all experiments were designed and powered to a minimum of 0.8 as calculated. Mice were allocated randomly into experimental groups. Owing to the nature of the cell culture experiments, randomization of the samples was not possible. As most studies were performed by individual researchers knowing the design of the studies, blinding during data collection and analysis was not performed.

All calculations, analysis and data plotting were performed using GraphPad Prism 6 or later, unless otherwise indicated. Differences between two independent samples were evaluated using two-tailed Student’s *t*-tests without adjustment for multiple comparisons, unless otherwise stated in the figure legends. Differences between multiple samples were analysed using one-way ANOVA with Tukey’s post hoc analysis, unless otherwise stated in the figure legends. No data were excluded from the analyses. *n* represents individual experiments from independently seeded cells or from different mice. All in vitro and in vivo assays were performed at least three times. The accompanying quantification and statistics were derived from at least *n* = 3 independent replicates. Data are represented as mean ± s.e.m. *P* values are stated in the figures.

### Reporting summary

Further information on research design is available in the Nature Portfolio Reporting Summary linked to this article.

## Online content

Any methods, additional references, Nature Portfolio reporting summaries, source data, extended data, supplementary information, acknowledgements, peer review information; details of author contributions and competing interests; and statements of data and code availability are available at 10.1038/s41556-023-01311-9.

### Supplementary information

Supplementary Information Supplementary Table 1

Reporting Summary

### Source data

Source Data Fig. 1 Statistical source data.

Source Data Figs. 1–6 Unprocessed western Blots for all main figures.

Source Data Fig. 2 Statistical source data.

Source Data Fig. 3 Statistical source data.

Source Data Fig. 4 Statistical source data.

Source Data Fig. 5 Statistical source data.

Source Data Fig. 6 Statistical source data.

Source Data Extended Data Fig./Table Statistical source data for Extended Data Figs. 1–4 and 6–9.

Source Data Extended Data Fig./Table Unprocessed western blots for Extended Data Figs. 1–3, 8 and 9.

## Data Availability

The proteomics datasets have been deposited in the ProteomeXchange database under accession number PXD046709. All other data supporting the findings of this study are available from the corresponding author on reasonable request. Source data are provided with this paper.
