# Supplementary material for: EPAC1 enhances brown fat growth and beige adipogenesis
Source: Nat Cell Biol. 2024 Jan 9;26(1):113–23. doi: 10.1038/s41556-023-01311-9 (PMC10791580; doi:10.1038/s41556-023-01311-9)
Supplement: Supplementary file 1 — Supplementary Table 1 [file 41556_2023_1311_MOESM1_ESM.pdf]

nature cell biology

Article

https://doi.org/10.1038/s41556-023-01311-9

EPAC1 enhances brown fat growth and beige adipogenesis

In the format provided by the authors and unedited

Gene name	Species	Primer sequence (5' – 3')	
Hprt	Mouse	*Forward*	GTCCCAGCGTCGTGATTAGC
		Reverse	TCATGACATCTCGAGCAAGTCTTT
Tnfa	Mouse	*Forward*	CCCTCACACTCAGATCATCTTCT
		Reverse	GCTACGACGTGGGCTACAG
Cxcl2	Mouse	*Forward*	CCCAGACAGAAGTCATAGCCAC
		Reverse	CTTCCGTTGAGGGACAGCAG
Arg1	Mouse	*Forward*	ACATTGGCTTGCGAGACGTA
		Reverse	ATCGGCCTTTTCTTCCTTCCC
Il10	Mouse	*Forward*	CCAAGGTGTCTACAAGGCCA
		Reverse	GCTCTGTCTAGGTCCTGGAGT
Ccl2	Mouse	*Forward*	TGGAGCATCCACGTGTTG
		Reverse	GCTGGTGAATGAGTAGCAGCA
Pparγ	Mouse	*Forward*	ACTGCAGCCCCCTATAGT
		Reverse	GGATCAGTTGGGTCAGTGGG
Ucp1	Mouse	*Forward*	TAAGCCGGCTGAGATCTTGT
		Reverse	GGCCTCTACGACTCAGTCCA
Nd5	Mouse	*Forward*	AGCATTCGGAAGCATCTTTG
		Reverse	TTGTGAGGACTGGAATGCTG
Cox8b	Mouse	*Forward*	GAACCATGAAGCCAACGACT
		Reverse	GCGAAGTTCACAGTGGTTCC
Tfam	Mouse	*Forward*	CCTTCGATTTTCCACAGAACA
		Reverse	GCTCACAGCTTCTTTGTATGCTT
Adbr3	Mouse	*Forward*	CCTTCAACCCGGTCATCTAC
		Reverse	GAAGATGGGGATCAAGCAAGC
Pdgfra	Mouse	*Forward*	TGTGCCCATTCGCAGGAAGAG
		Reverse	TTGGCCACCTTGACACTGCG
Epac1	Mouse	*Forward*	CTGGACACCACTTACCAACA
		Reverse	ATTTTTGTGTCTCGGATGAGG
Epac2	Mouse	*Forward*	CGCCATGCAACCATCGTTACC
		Reverse	GAGCCCGTTTCCATAACACC
Fabp4	Mouse	*Forward*	TGAAAGAAGTGGGAGTGGGCTTTGC
		Reverse	CACCACCAGCTTGTCACCATCTCGT
UCP1	human	*Forward*	TGCCCAACTGTGCAATGA A
		Reverse	CCAGGATCCAAGTCGCAAGA
PGC1A	human	*Forward*	CTGTGTCACCACCCAAATCCTTAT
		Reverse	TGTGTCGAGAAAAGGACCTTG A
PPARG	human	*Forward*	AGCCTCATGAAGAGCCTTCCA
		Reverse	TCCGGAAGAAACCCTTGCA
CEBPB	human	*Forward*	ATGGATTTAAAGGCAGGCGG
		Reverse	GTTCATGCAACGCCTGGT
GAPDH	human	*Forward*	TGGTCTCCTCTGACTTCAAC
		Reverse	GTGAGGGTCTCTCTCTTCCT
EPAC1	human	*Forward*	GCTGCTCTGGCCGGGA
		Reverse	GAATTGGGCATCTCGGTCCT
FABP4	human	*Forward*	TGTGCAGAAATGGGATGGAAA
		Reverse	CAACGTCCCTTGGCTTATGCT
