# Supplementary material for: EPAC1 enhances brown fat growth and beige adipogenesis
Source: Nat Cell Biol. 2024 Jan 9;26(1):113–23. doi: 10.1038/s41556-023-01311-9 (PMC10791580; doi:10.1038/s41556-023-01311-9)
Supplement: Supplementary file 4 — Unprocessed western Blots for all main figures. [file 41556_2023_1311_MOESM4_ESM.pdf]

Source Data
Main Figures
Uncropped blots

Reverte-Salisa, L. *et al*

Fig 1 a

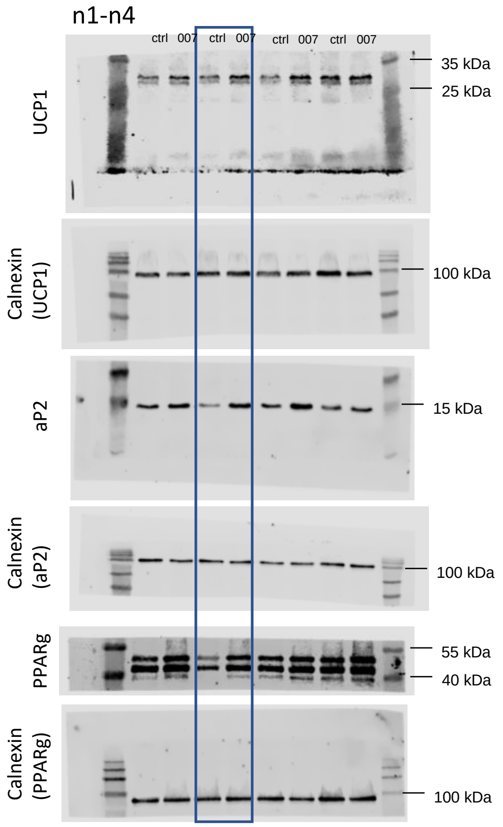

Fig 1 b

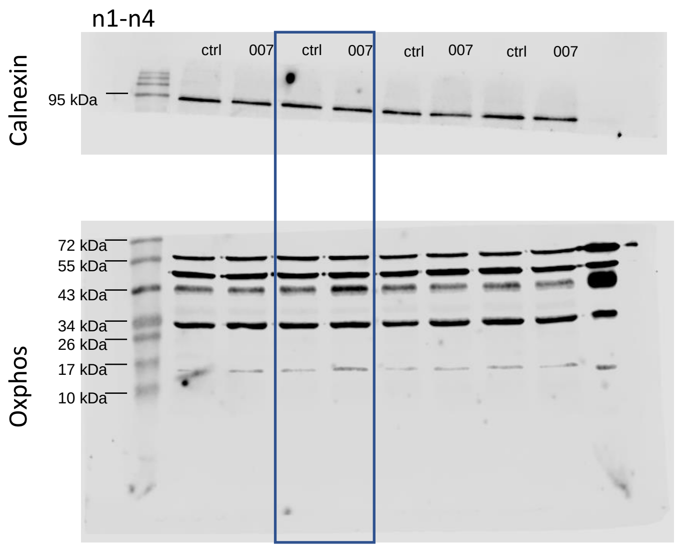

Fig 1 g

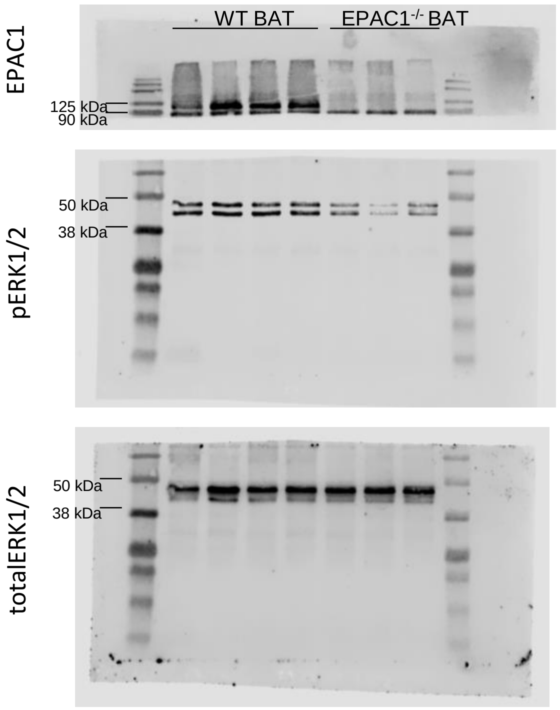

Fig 1 i

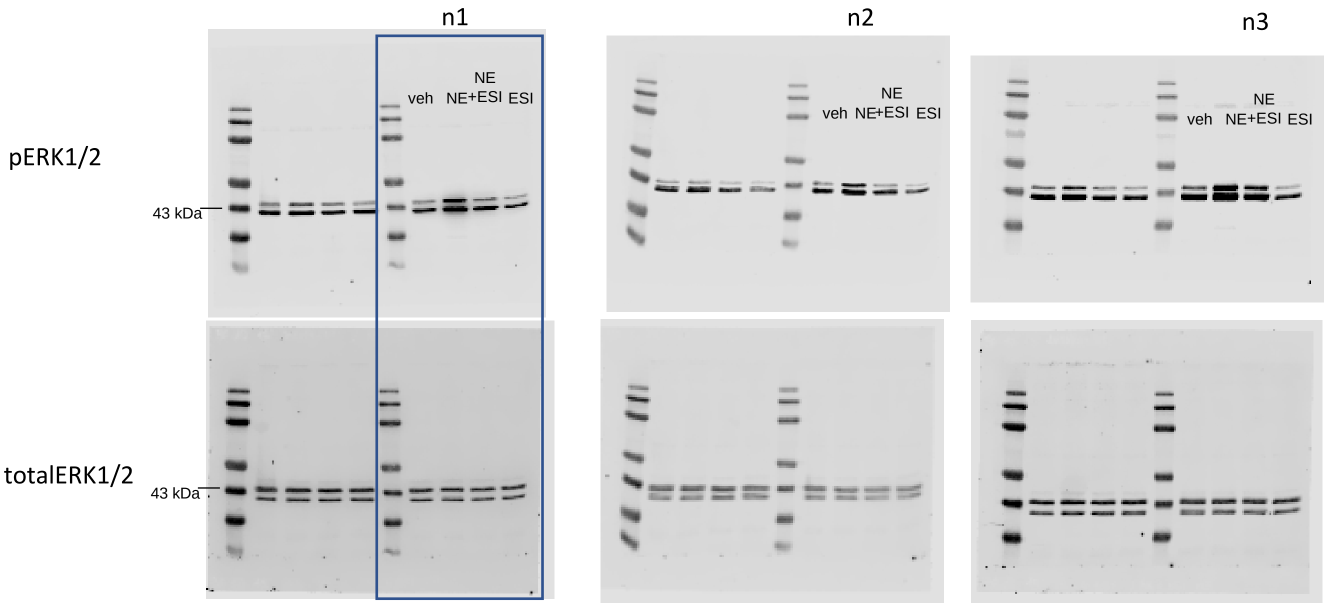
n1
n2
n3
pERK1/2
totalERK1/2
43 kDa
veh
NE
NE+ESI
ESI

Fig 1k

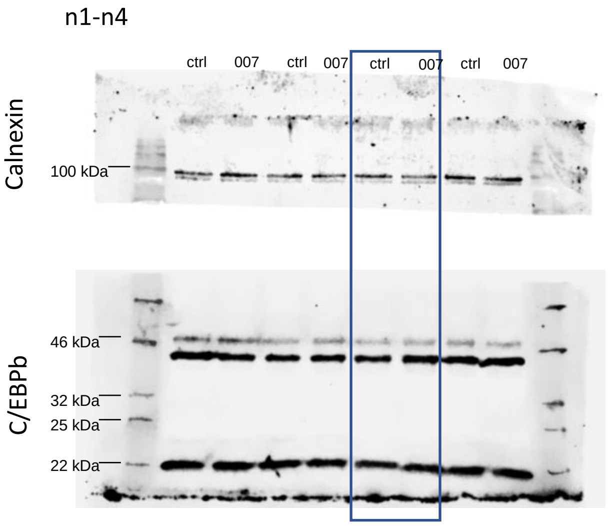
n1-n4
ctrl
007
Calnexin
C/EBPb
100 kDa
46 kDa
32 kDa
25 kDa
22 kDa

Fig 2c

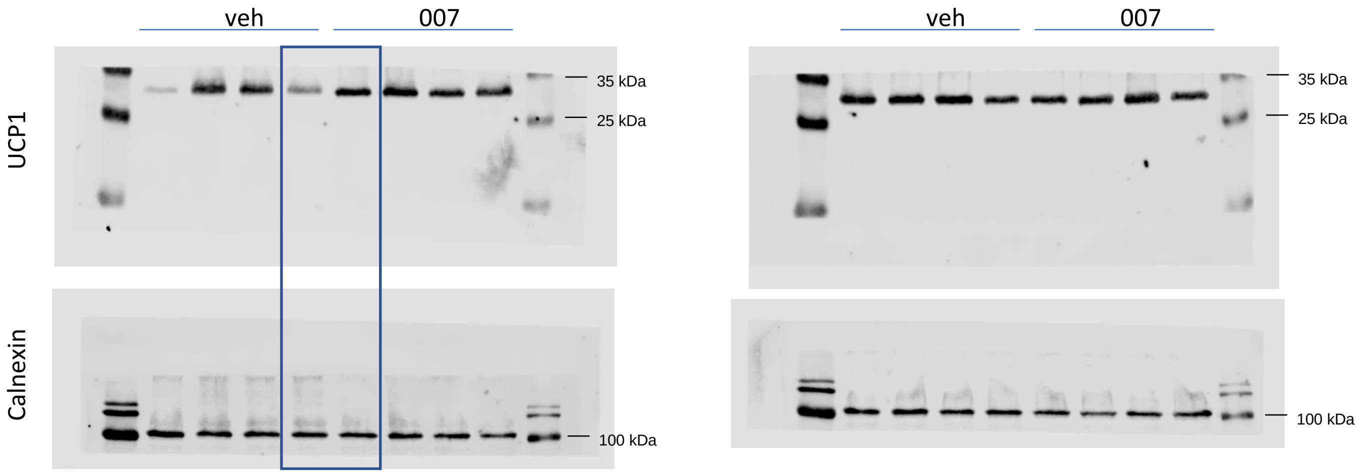

veh
007
veh
007
UCP1
Calnexin
35 kDa
25 kDa
100 kDa
35 kDa
25 kDa
100 kDa

Fig 2h

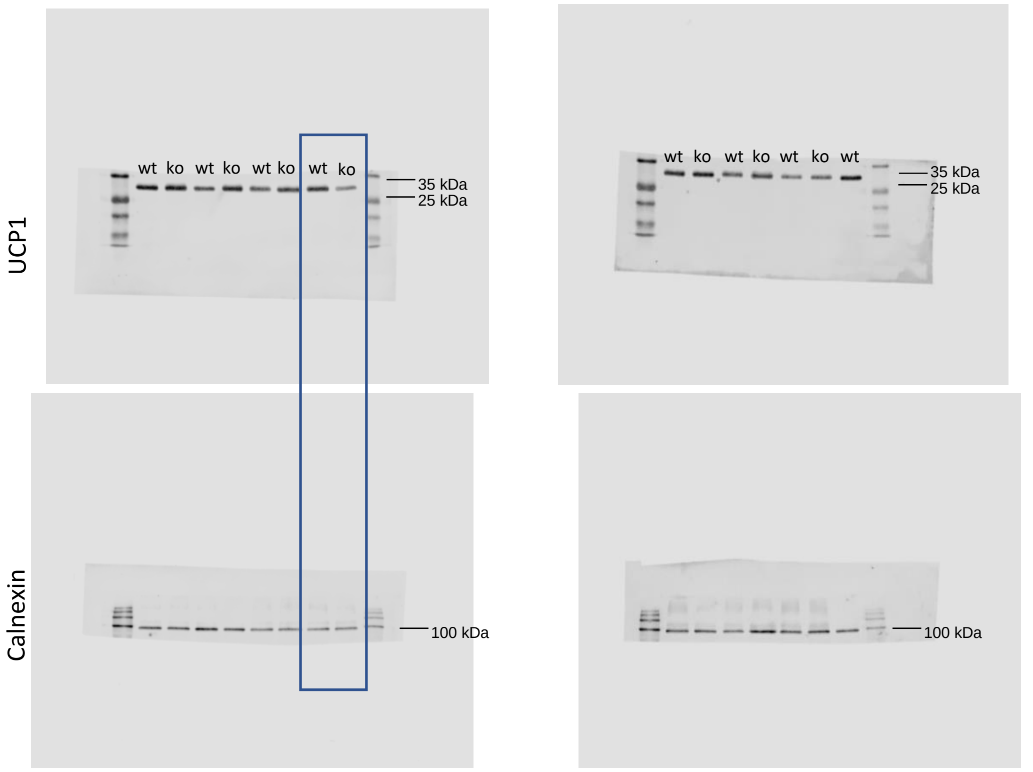

wt ko wt ko wt ko wt ko
35 kDa
25 kDa
wt ko wt ko wt ko wt
35 kDa
25 kDa
UCP1
Calnexin
100 kDa
100 kDa

Fig 3g

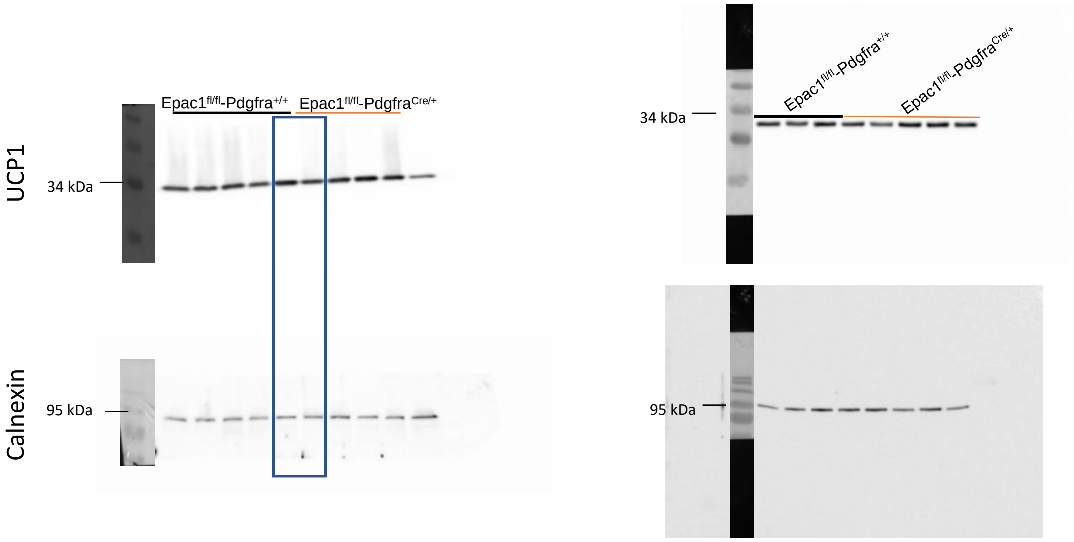

UCP1
34 kDa
Epac1fl/fl-Pdgfra+/+
Epac1fl/fl-PdgfraCre/+
Calnexin
95 kDa
34 kDa
Epac1fl/fl-Pdgfra+/+
Epac1fl/fl-PdgfraCre/+
95 kDa

Fig 4e

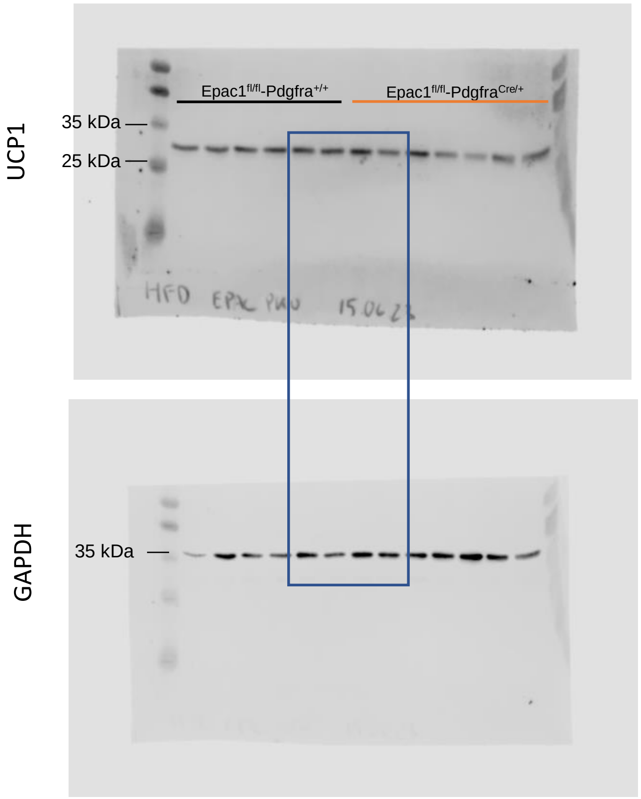

UCP1
35 kDa
25 kDa
Epac1fl/fl-Pdgfra+/+
Epac1fl/fl-PdgfraCre/+
GAPDH
35 kDa
