# Supplementary material for: EPAC1 enhances brown fat growth and beige adipogenesis
Source: Nat Cell Biol. 2024 Jan 9;26(1):113–23. doi: 10.1038/s41556-023-01311-9 (PMC10791580; doi:10.1038/s41556-023-01311-9)

Source Data
Extended Data Figures
Uncropped blots

Reverte-Salisa, L. *et al*

Extended Data Fig 1b

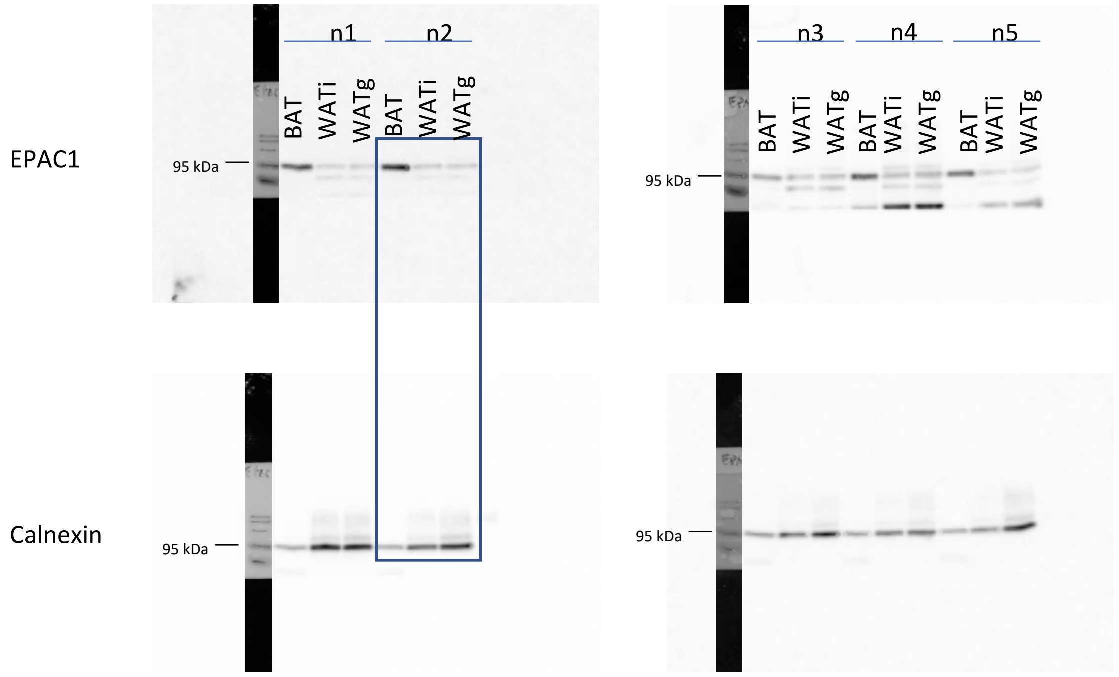

Extended Data Fig 2d

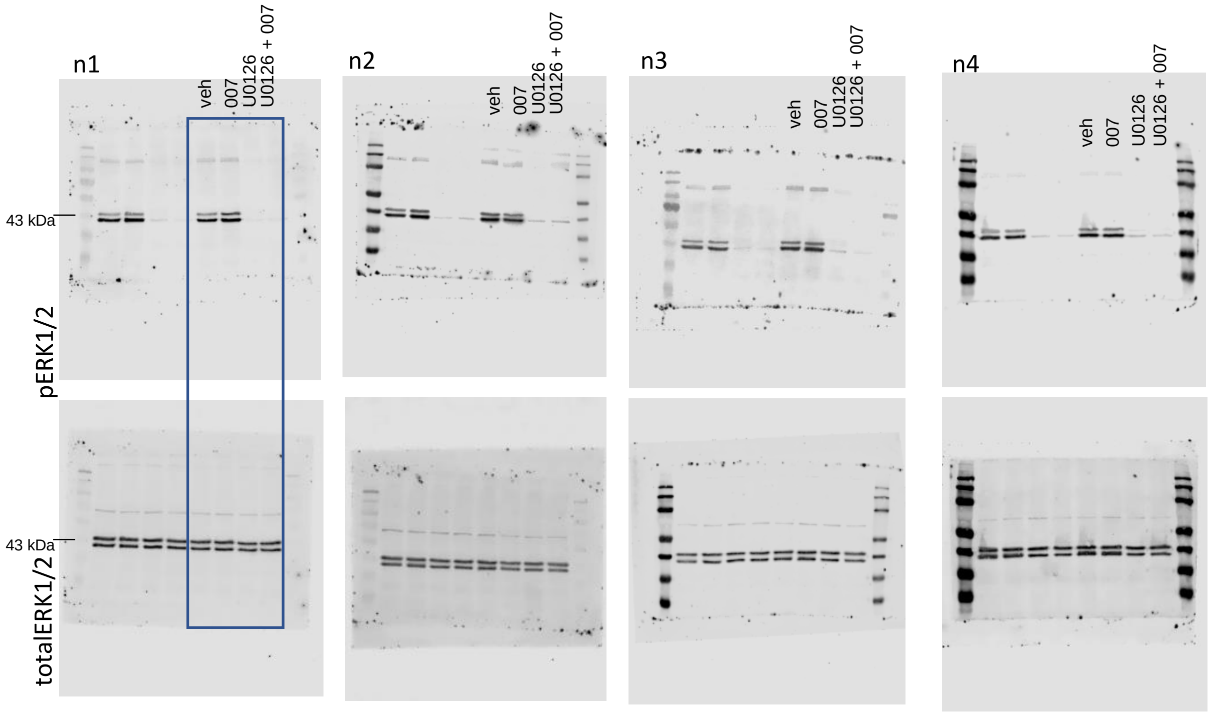

Extended Data Fig 2e

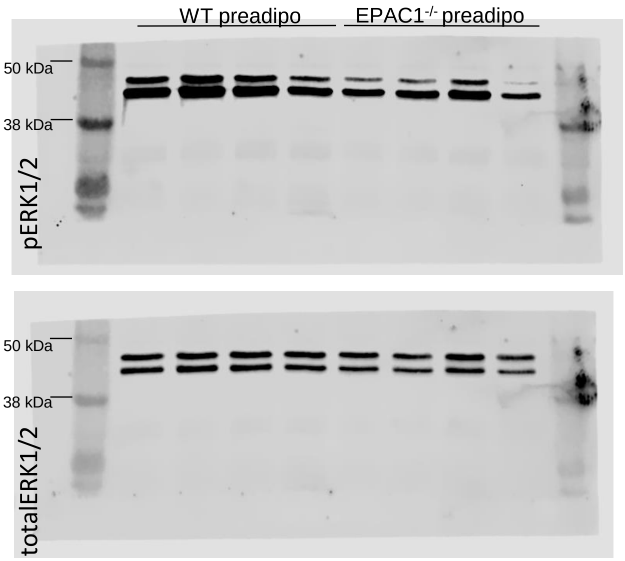

Extended Data Fig 2g

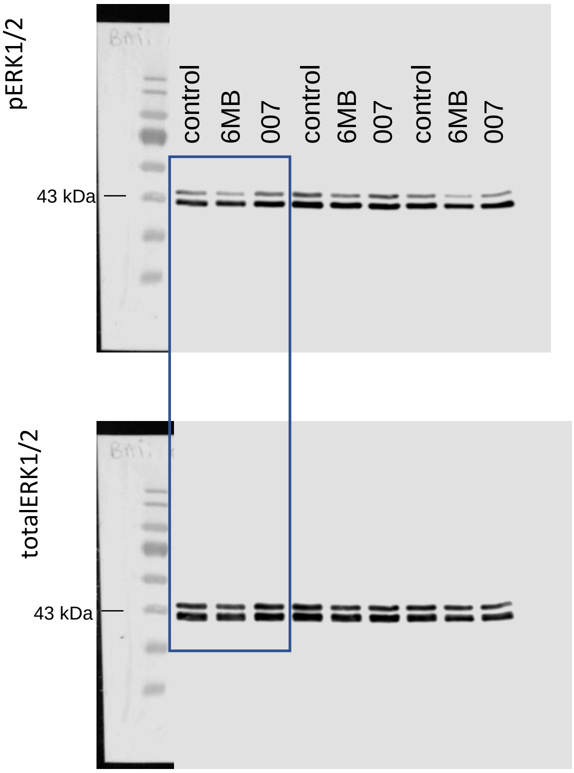

Extended Data Fig 2o

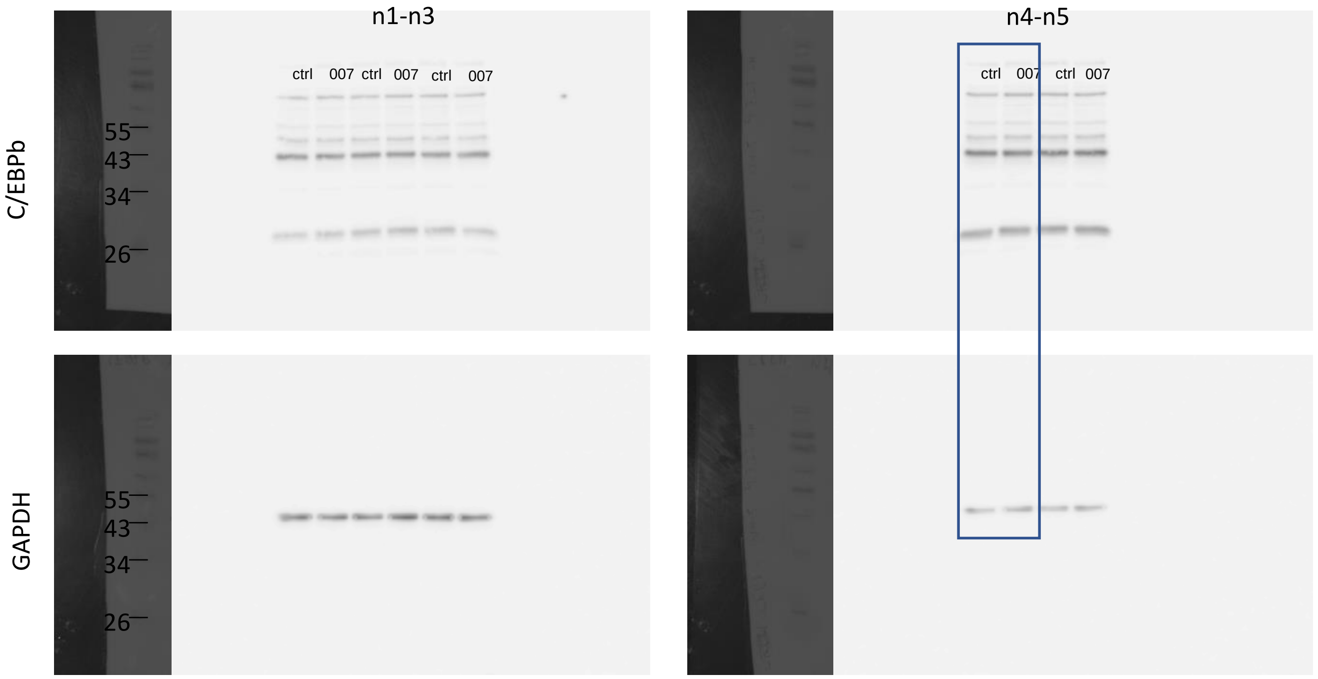

Extended Data Fig 2p

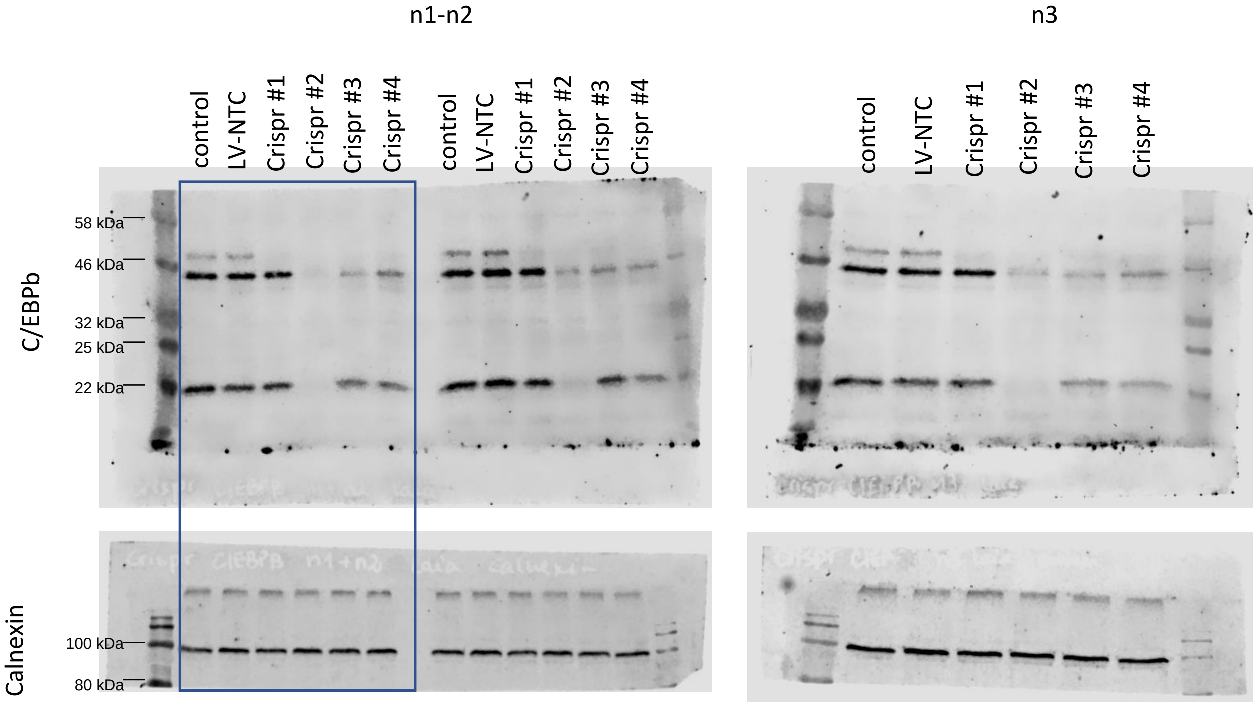

Extended Data Fig 2s

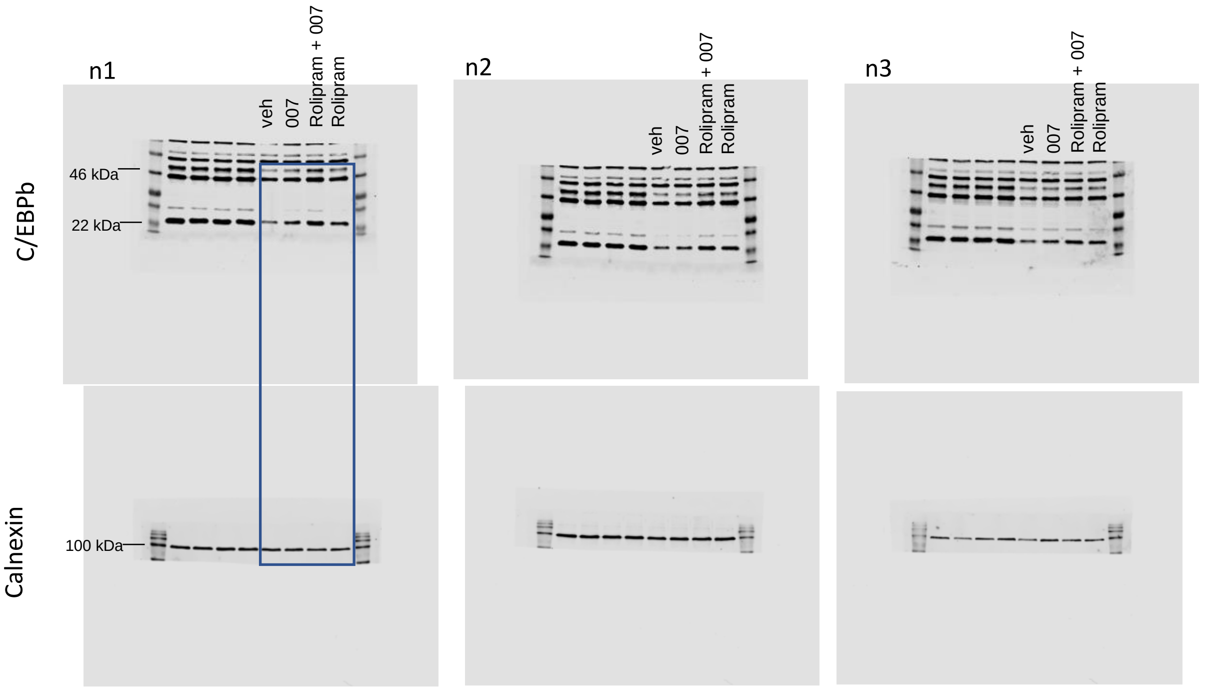

Extended Data Fig 2t

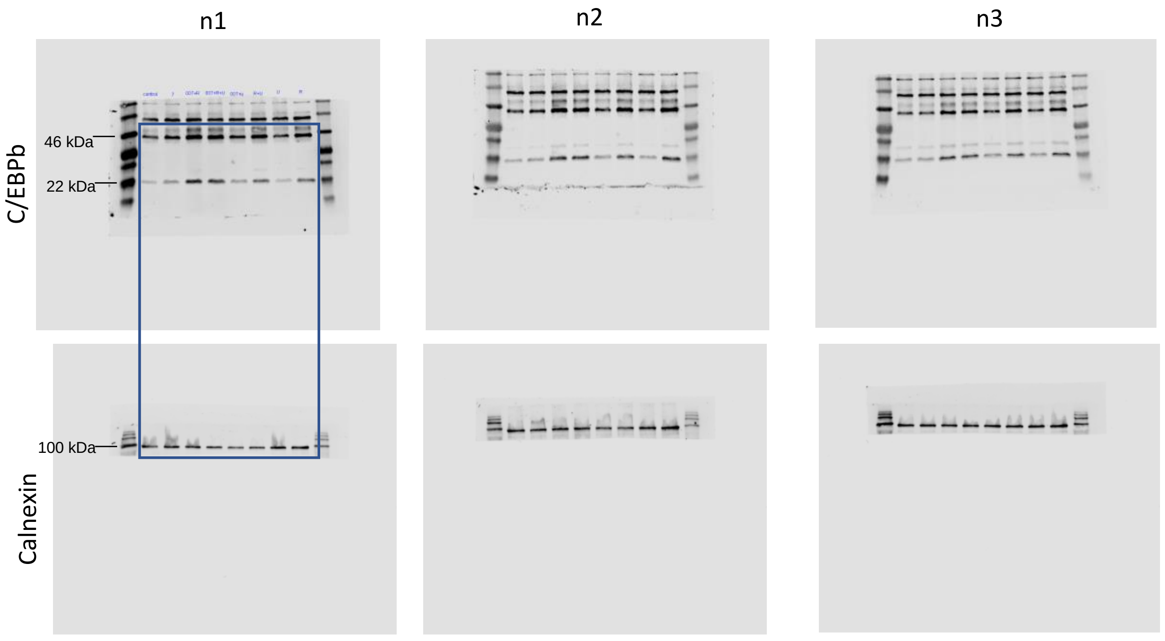

Extended Data Fig 3e

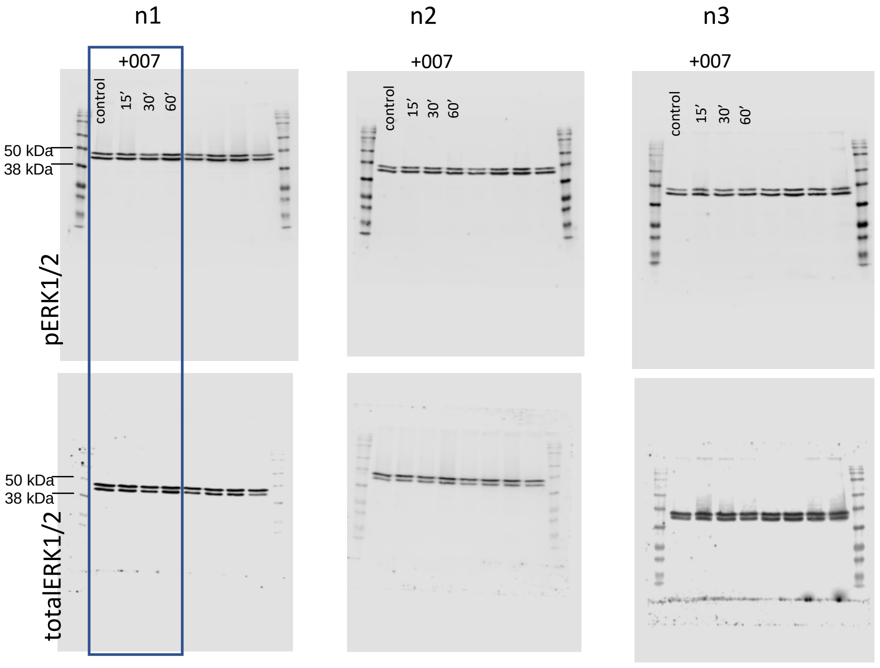

Extended Data Fig 3g

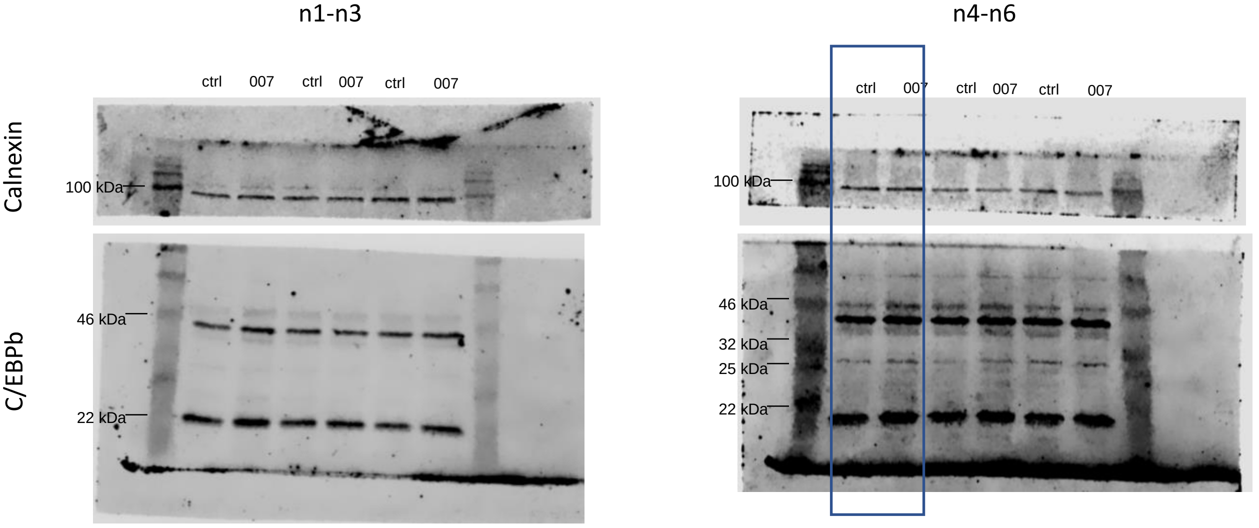

Extended Data Fig 8e and 8j

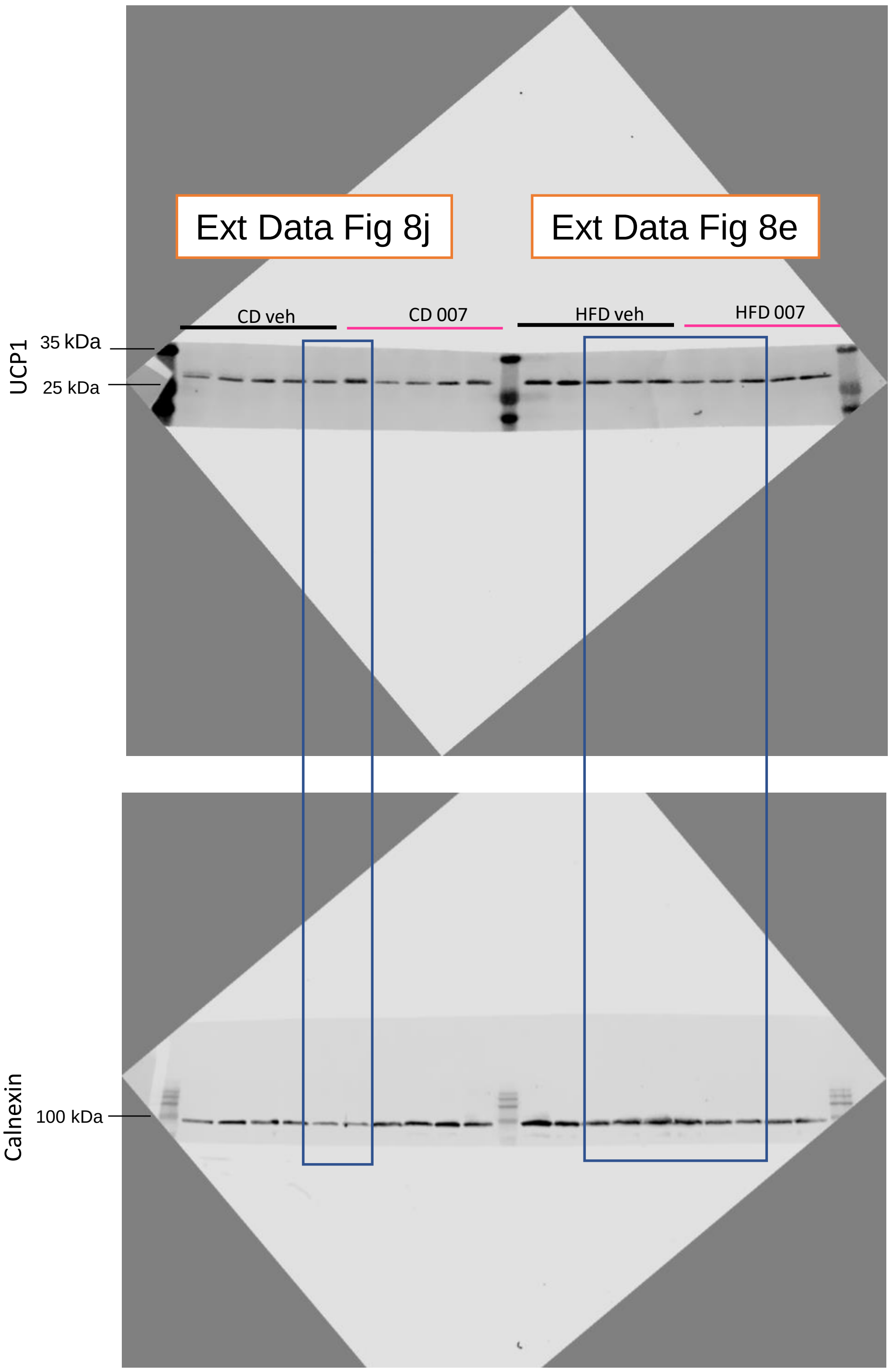

Extended Data Fig 9a

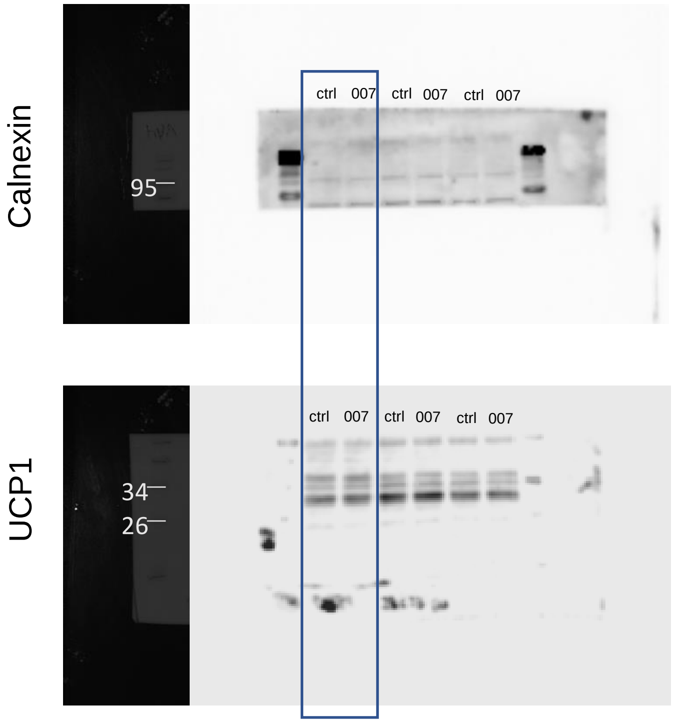

Supplement: Supplementary file 11 — Unprocessed western blots for Extended Data Figs. 1–3, 8 and 9. [file 41556_2023_1311_MOESM11_ESM.pdf]
